# Impact of oral bacterial lysates on asthma control and immune parameters in children: evidence from an updated systematic review and meta-analysis of randomized trials

**DOI:** 10.3389/fphar.2026.1801312

**Published:** 2026-06-11

**Authors:** Yifeng Chen, Lingling Shi, Yaqin Wang, Lihua Chen, Lizhen Wang, Yanfen Ying

**Affiliations:** Taizhou Hospital of Zhejiang Province Affiliated to Wenzhou Medical University, Taizhou, China

**Keywords:** bacterial lysates, immunomodulation, meta-analysis, OM-85, pediatric asthma

## Abstract

**Background:**

The role of oral bacterial lysates (OBLs) as adjuvant immunomodulatory therapy in pediatric asthma requires clarification. This systematic review and meta-analysis evaluates their efficacy and safety.

**Methods:**

We searched eight databases for randomized controlled trials (RCTs) in children comparing standard asthma therapy plus OBLs versus standard therapy/placebo. Primary outcomes were clinical (wheezing/infection frequency, symptom improvement time, treatment efficacy) and lung function parameters. Secondary outcomes included immune biomarkers and adverse events. Random-effects meta-analyses were performed.

**Results:**

Twenty-eight RCTs (n = 2,893) were included. Adjunctive OBLs therapy significantly reduced wheezing/exacerbation frequency (Mean Difference (MD) = −3.00,95% confidence intervals (CI): 4.07 to −1.93), shortened symptom improvement time (MD = −3.13 days, 95%CI: 4.10 to −2.15), reduced Respiratory Tract Infection (RTI) frequency (MD = −2.43,95%CI: 3.62 to −1.23) and increased overall treatment efficacy rate (relative rates (RR) = 1.17, 95%CI: 1.13–1.21). Improvements occurred in lung function (Forced Expiratory Volume in 1 s [FEV1], Forced Vital Capacity [FVC], Peak Expiratory Flow [PEF]) and immune parameters (increased the level of T-lymphocyte subsets (CD3+,CD4+,CD4+/CD8+) and salivary secretory immunoglobulin A (sIgA), decreased peripheral eosinophil (EOS) count and the level of interleukin-4(IL-4), eosinophil cationic protein (ECP), fractional exhaled nitric oxide (FeNO). Adverse events did not increase significantly (RR = 1.26, 95%CI: 0.93–1.70). Subgroup analyses showed consistent benefits across follow-up duration, age, and sample size, with background inhaled corticosteroids (ICS) therapy being a potential effect modifier (P = 0.06).

**Conclusion:**

Adjunctive OBLs therapy is associated with improved clinical outcomes, lung function, and immune modulation in pediatric asthma, with a favorable safety profile. However, methodological limitations, substantial heterogeneity, and potential biases warrant caution. While promising, more rigorous and long-term trials are needed to define its precise therapeutic role and target population.

**Systematic Review Registration:**

Identifier CRD420261281796.

## 1 Introduction

Asthma remains the most prevalent chronic respiratory disease in children worldwide, constituting a significant global public health challenge characterized by recurrent episodes of wheezing, breathlessness, chest tightness, and cough (Bateman et al., 2008). These symptoms stem from a complex interplay of chronic airway inflammation, bronchial hyperresponsiveness, and variable airflow obstruction (Barnes, 2008). The global burden is substantial, with an estimated 5%–10% of pediatric populations affected, leading to frequent healthcare utilization, diminished quality of life, impaired school performance, and considerable economic costs for families and healthcare systems (Asher and Pearce, 2014).

The cornerstone of pediatric asthma management involves controller medications aimed at suppressing underlying inflammation and preventing exacerbations. Inhaled corticosteroids (ICS) are the first-line and most effective anti-inflammatory controller therapy (Sanjiv-Sin gh Rawat. Global Initiative for Asthma GINA, 2025). Leukotriene receptor antagonists (LTRA) and long-acting beta2-agonists (LABA), often in combination with ICS, serve as additional options (Muireann et al., 2009). While these therapies are effective for many, clinical challenges persist. A subset of children continues to experience breakthrough symptoms, acute exacerbations often triggered by viral respiratory infections, and a phenomenon described as the “recurrent wheeze-infection cycle” (Holt and Sly, 2012; Jartti and Gern, 2017). Furthermore, concerns regarding the potential long-term effects of ICS, though generally considered safe, and suboptimal adherence to inhalation devices can limit real-world effectiveness (Petrisko et al., 2008; Marjolein et al., 2014). These limitations highlight the need for complementary therapeutic strategies that target different aspects of asthma pathophysiology, particularly the immune dysregulation and susceptibility to infections that drive disease morbidity.

The pathogenesis of asthma, especially in children, involves a dysregulated immune response. A shift towards T-helper 2 (Th2) cell-dominant inflammation is common, leading to elevated levels of cytokines like interleukin-4 (IL-4), IL-5, and IL-13, which promote eosinophilic inflammation, immunoglobulin E (IgE) production, and airway remodeling (Gans Melissa and Gavrilova, 2019). Concurrently, impaired innate immune responses and mucosal defense mechanisms are believed to increase susceptibility to respiratory pathogens, which are the most common triggers of acute exacerbations in children (Busse et al., 2010). This intersection between allergic inflammation and infection susceptibility provides a rationale for immunomodulatory interventions.

Early life is crucial for establishing immune tolerance, which is important for maintaining the stability and normal physiological functions of innate and adaptive immune responses. Stable immune function in the airways depends on appropriate interactions among the microbiota colonizing the mucosa, the host immune response, and environmental microorganisms (Bloomfield Sally et al., 2016). Urbanization has significantly improved hygiene conditions and changed daily life patterns. The increased rate of cesarean sections, the decline in breastfeeding rates, and the overuse of antibiotics may significantly reduce microbial diversity, leading to immune tolerance dysfunction and promoting the occurrence of allergic diseases such as asthma (Adnan et al., 2012). Based on these theoretical backgrounds, the impact of environmental microbial components on immune system function has received increasing attention. Subsequently, a large number of animal and clinical trials have been conducted to study microorganisms and their lysates or metabolites. Bacterial lysates are oral immunomodulators prepared from extracts of inactivated bacteria commonly involved in respiratory tract infections (e.g., *Haemophilus* influenzae, *Streptococcus* pneumoniae, *Klebsiella pneumoniae*, etc.). Products like OM-85 (Broncho-Vaxom) have been extensively studied. Their proposed mechanism of action involves stimulating both innate and adaptive immunity via the gut-associated lymphoid tissue (Vadim et al., 2021; Adriana and Chorostowska-Wynimko, 2008). They are thought to enhance mucosal defense by increasing sIgA production, promote a balanced Th1/Th2 response by modulating cytokine production (e.g., increasing interferon-γ (IFN-γ), decreasing IL-4), and potentially reduce the frequency and severity of respiratory infections (Agnieszka et al., 2022; Elpida et al., 2025). By potentially breaking the “infection-wheeze” cycle and modulating the underlying inflammatory milieu, bacterial lysates present a promising non-steroidal adjuvant therapy for pediatric asthma.

Over the past 2 decades, numerous randomized controlled trials (RCTs), particularly from regions like China and Europe, have investigated the add-on effect of bacterial lysates to standard asthma therapy in children. These studies have reported outcomes across diverse domains: clinical (e.g., exacerbation rate, symptom recovery time), functional (lung function), immunological (T-cell subsets, cytokines), and safety. However, the results have been inconsistent. Some trials report significant benefits in reducing wheezing episodes and improving immune parameters, while others show more modest or non-significant effects (Lan et al., 2022; Razi et al., 2010; Zhuang-Gui et al., 2007; Emeryk et al., 2018; Siri et al., 2020). Furthermore, existing systematic reviews on this topic are either limited in scope, outdated, or focused on specific subsets of patients (e.g., those with recurrent infections), leaving a gap in the evidence synthesis (Castro-Rodriguez et al., 2024).

A comprehensive and up-to-date quantitative synthesis is therefore urgently needed to clarify the overall value of this adjunctive therapy. Specifically, there is a need to concurrently evaluate its core clinical benefits (on exacerbations, symptoms, and infections), its objective physiological impact (on lung function), its immunomodulatory effects, and its safety profile within a single analytical framework. Such an analysis would provide clinicians and guideline developers with a clearer, evidence-based perspective on the role of bacterial lysates in the management of pediatric asthma.

Therefore, we conducted this systematic review and meta-analysis of RCTs to critically appraise and synthesize the existing evidence regarding the efficacy and safety of bacterial lysates as an adjuvant therapy in children with asthma. Our primary objectives were to determine their effects on: 1) key clinical outcomes (wheezing/exacerbation frequency, time to symptom improvement, RTI frequency, overall treatment efficacy rate); 2) lung function (FEV1, FVC, PEF). Secondary objectives were to assess their impact on: 3) immunological and inflammatory biomarkers (levels of T-lymphocyte subsets, serum cytokine levels, immunoglobulin levels in serum, FeNO levels, peripheral EOS numbers, ECP levels in serum); and 4) the incidence of adverse events.

## 2 Materials and methods

### 2.1 Protocol and registration

This systematic review and meta-analysis was conducted and reported in accordance with the Preferred Reporting Items for Systematic Reviews and Meta-Analyses (PRISMA) guidelines (Page et al., 2021). A pre-defined protocol for this review was registered, the registration number is CRD420261281796.

### 2.2 Eligibility criteria

#### 2.2.1 Types of studies

We included all published randomized controlled trials (RCTs) that evaluated the adjuvant use of bacterial lysates in children with asthma. RCTs were eligible regardless of their blinding status (open-label, single-blind, or double-blind) or publication language (Chinese or English). Studies with non-randomized designs (e.g., retrospective studies, cohort studies), review articles, meta-analyses, animal studies, case reports, and editorials were excluded.

#### 2.2.2 Types of participants

The population of interest comprised children and adolescents under 18 years of age with a physician-diagnosis of bronchial asthma, based on the diagnostic criteria applied in each original study. Studies involving participants with other primary respiratory conditions (e.g., isolated allergic rhinitis, cystic fibrosis) were excluded.

#### 2.2.3 Types of interventions

The intervention group was required to receive standard asthma therapy plus an adjunctive oral bacterial lysate. The primary bacterial lysates of interest were OM-85 (Broncho-Vaxom, also known as “FanFuShu”) and Polyvalent Mechanical Bacterial Lysates (PMBL, Ismigen). The control group received standard asthma therapy alone or an identical placebo. Standard asthma therapy was defined according to contemporary guidelines and could include, but was not limited to: inhaled corticosteroids (ICS), short or long-acting beta-2 agonists (SABA/LABA), leukotriene receptor antagonists (e.g., montelukast), systemic corticosteroids, or combinations thereof.

#### 2.2.4 Types of outcome measures

Outcomes were categorized as primary or secondary.

Primary Outcomes focused on clinical efficacy and lung function:

Wheezing/Exacerbation Frequency: Mean number of wheezing episodes or asthma exacerbations during treatment and a specified follow-up period.

Time to Symptom Improvement: Mean duration (in days) for the improvement of cough and wheezing from treatment initiation.

Respiratory Tract Infection (RTI) Frequency: Mean number of acute RTI episodes during follow-up.

Overall Treatment Efficacy Rate: The proportion of patients categorized as “effective” or “markedly effective” based on composite clinical criteria defined in each original study (a dichotomous outcome).

Lung Function Parameters: Absolute post-treatment values of Forced Expiratory Volume in 1 s (FEV1, L), Forced Vital Capacity (FVC, L), and Peak Expiratory Flow (PEF, L/s).

Secondary Outcomes included immunological, inflammatory, and safety parameters:

Immunological Biomarkers: Peripheral blood levels of T-lymphocyte subsets (CD3+%, CD4+%, CD4+/CD8+ ratio), serum cytokines (Interferon-γ [IFN-γ], Interleukin-4 [IL-4], Interleukin-10 [IL-10]), serum eosinophil cationic protein (ECP), and peripheral eosinophil (EOS) count.

Immunoglobulin Levels: Serum levels of Immunoglobulin M (IgM) and G (IgG), salivary secretory Immunoglobulin A (sIgA), and total serum Immunoglobulin E (IgE).

Airway Inflammation Biomarker: Fractional exhaled Nitric Oxide (FeNO, ppb).

Safety: Incidence of all reported adverse events (AEs), such as gastrointestinal symptoms (abdominal pain, nausea, vomiting, diarrhea), drowsiness, dizziness, and skin rash.

#### 2.2.5 Exclusion criteria

Studies were excluded if they met any of the following criteria: (1) published in languages other than Chinese or English; (2) duplicate publications (the most complete version was retained); (3) lacked relevant primary or secondary outcome data as defined above; (4) had a total sample size of less than 60 participants; (5) presented incomplete, missing, or non-extractable data for meta-analysis; (6) the full-text article could not be obtained.

### 2.3 Information sources and search strategy

A comprehensive and systematic literature search was performed across eight electronic databases from their inception to 28 February 2025: PubMed, EMBASE, the Cochrane Central Register of Controlled Trials (CENTRAL), Web of Science Core Collection, China National Knowledge Infrastructure (CNKI), WanFang Data, Chinese Biomedical Literature Database (CBM), and VIP Database for Chinese Technical Periodicals (VIP). The search strategy employed a combination of controlled vocabulary (e.g., MeSH in PubMed, Emtree in EMBASE) and free-text keywords related to the population (“asthma”, “child”) and the intervention (“bacterial lysates”, “OM-85″, “Broncho-Vaxom”). The search syntax was adapted for each database. No filters for date or language were applied. The reference lists of all included studies and relevant review articles were manually screened to identify any additional eligible publications. An example of the full search strategy for PubMed is provided in Supplementary Material.

### 2.4 Study selection process

Records retrieved from all databases were imported into EndNote X9 (Clarivate Analytics) for deduplication. The study selection process was conducted independently by two reviewers. Initially, titles and abstracts were screened against the eligibility criteria. Subsequently, the full texts of potentially relevant articles were obtained and assessed in detail. Any disagreements between the two reviewers at any stage of the selection process were resolved through discussion or, if necessary, by arbitration from a third senior reviewer. The study selection process, including the number of records identified, excluded, and included, was documented using a PRISMA flow diagram.

### 2.5 Data extraction and management

A standardized, pre-piloted data extraction form was developed in Microsoft Excel. Two reviewers independently extracted the following data from each included study:

Study Identification and Characteristics: First author, year of publication, journal, country where the study was conducted, study design.

Participant Characteristics: Diagnostic criteria for asthma, total sample size, number of participants randomized to intervention and control groups, age (mean ± standard deviation [SD]), gender distribution.

Intervention Details: Type, dosage, and regimen of the bacterial lysate; detailed description of the concomitant standard therapy in both groups; treatment duration and follow-up period.

Outcome Data: For continuous outcomes, the post-treatment mean, SD, and sample size for both groups were extracted. For dichotomous outcomes, the number of events and the total number of participants in each group were extracted. When data were presented only graphically, WebPlotDigitizer (Version 4.6) was used to extract numerical values. For outcomes reported as median and interquartile range (IQR), estimates of mean and SD were derived using established conversion formulae (Xiang et al., 2014).

Information for Risk of Bias Assessment: Two reviewers independently assessed the methodological quality of each included study. Details pertinent to judging the risk of bias were extracted according to the Cochrane Risk of Bias tool, covering domains such as random sequence generation and allocation concealment. Any discrepancies were resolved through discussion or by a third reviewer.

Any discrepancies in extracted data were resolved by consensus after re-checking the original article. Corresponding authors were not contacted for missing data.

### 2.6 Data synthesis and statistical analysis

Statistical analyses were performed using Review Manager (RevMan, version 5.4, The Cochrane Collaboration) and Stata (version MP 16.0, StataCorp). A two-tailed P-value <0.05 was considered statistically significant for all tests except where otherwise specified for heterogeneity.

Measures of Treatment Effect: For dichotomous outcomes (e.g., treatment efficacy, adverse events), the pooled treatment effect was expressed as a Risk Ratio (RR) with a 95% Confidence Interval (CI). For continuous outcomes measured on the same scale (e.g., FEV1 in liters), the Mean Difference (MD) with 95% CI was calculated. For continuous outcomes measured with different scales or units (e.g., cytokine levels), the Standardized Mean Difference (SMD) with 95% CI was used.

Assessment of Heterogeneity: Statistical heterogeneity across studies was assessed using the Cochran’s Q chi-squared test (with a significance level of P < 0.10 indicating significant heterogeneity) and quantified using the I^2^ statistic (Higgins, 2011). I^2^ values of approximately 25%, 50%, and 75% were interpreted as indicating low, moderate, and high heterogeneity, respectively (Higgins Julian et al., 2003).

Data Synthesis and Model Selection: Meta-analysis was performed only when three or more studies reported the same outcome. If no substantial heterogeneity was detected (I^2^ < 50% and P for Q test >0.10), a fixed-effect model (Mantel-Haenszel method for dichotomous data, inverse-variance method for continuous data) was applied. Otherwise, a random-effects model (DerSimonian and Laird method) was used.

Subgroup and Sensitivity Analyses: Pre-specified subgroup analyses were conducted for the primary outcomes of wheezing frequency and time to symptom improvement to explore potential sources of heterogeneity. Subgroups were defined by: (1) use of ICS in the control regimen (Yes vs. No/Placebo), (2) follow-up duration (<12 months vs. ≥12 months), (3) participant age (≤5 years vs. >5 years), and (4) study sample size (<100 vs. ≥100). Sensitivity analysis was performed using the “leave-one-out” method, sequentially removing each study to assess its impact on the overall pooled estimate and heterogeneity.

Assessment of Reporting Biases: Publication bias was assessed using a combination of graphical and statistical methods. First, funnel plots were visually inspected for asymmetry. Then, for outcomes pooled from ten or more studies, statistical tests were performed, including Begg’s rank correlation test and Egger’s linear regression test (Jartti and Gern, 2017; Sterne et al., 2011). A P-value <0.10 in these tests was considered suggestive of statistically significant asymmetry, which could indicate the presence of publication bias or other small-study effects.

### 2.7 Risk of bias assessment in individual studies

The methodological quality of each included RCT was independently assessed by two reviewers using the original Cochrane Collaboration’s ‘Risk of Bias’ tool (RoB 1.0) (Higgins Julian et al., 2011). This tool evaluates six domains: random sequence generation, allocation concealment, blinding of participants and personnel, blinding of outcome assessment, incomplete outcome data, and selective reporting. Each domain was judged as having a “low,” “high,” or “unclear” risk of bias. Disagreements were resolved by consensus. The overall risk of bias for each study was summarized. The results of the assessment were presented graphically using the robvis package in R software.

## 3 Results

### 3.1 Study selection

**FIGURE 1:**
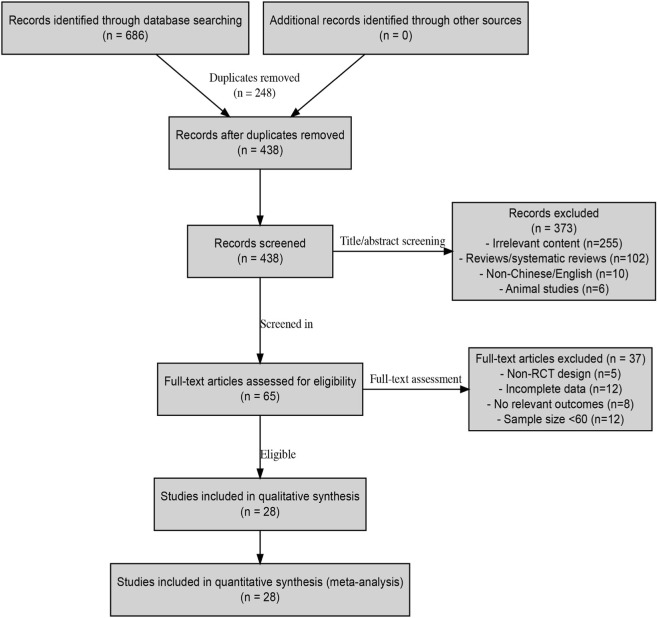
PRISMA flow diagram of study selection process. Flow diagram illustrating the study selection process according to the Preferred Reporting Items for Systematic Reviews and Meta-Analyses (PRISMA) guidelines. A total of 686 records were identified from eight databases (CBM, CNKI, VIP, WanFang, PubMed, EMBASE, Web of Science, Cochrane Library). After removing 248 duplicates, 438 records were screened by title and abstract. Of these, 373 records were excluded (irrelevant content: n = 255; review articles: n = 102; non-Chinese/non-English publications: n = 10; animal studies: n = 6). The remaining 65 full-text articles were assessed for eligibility. A further 37 articles were excluded for the following reasons: non-RCT design (n = 5); incomplete or non-extractable data (n = 12); absence of relevant outcome measures (n = 8); sample size <60 participants (n = 12). Ultimately, 28 randomized controlled trials (RCTs) were included in the qualitative synthesis and quantitative meta-analysis.

A total of 686 records were initially identified through database searching: CBM (n = 89), CNKI (n = 93), VIP (n = 16), WanFang (n = 110), PubMed (n = 31), EMBASE (n = 237), Web of Science (n = 81), and the Cochrane Library (n = 29). No additional records were identified from other sources. After removing 248 duplicates, 438 records remained for title and abstract screening. Of these, 373 records were excluded for the following reasons: irrelevant research content (n = 255), review/systematic review (n = 102), non-Chinese/non-English publication (n = 10), and animal studies (n = 6). The full texts of the remaining 65 articles were assessed for eligibility. Subsequently, 37 articles were excluded due to: inability to obtain full text (n = 0), sample size <60 (n = 12), absence of relevant outcome measures (n = 8), incomplete or non-extractable data (n = 12), and non-RCT study design (n = 5). Ultimately, 28 randomized controlled trials (RCTs) were included in the qualitative and quantitative synthesis (meta-analysis). The detailed study selection process is illustrated in the PRISMA flow diagram (Figure 1).

### 3.2 Characteristics of included studies

The 28 included RCTs were published between 2010 and 2024. The sample sizes ranged from 61 to 200 participants, with a total of 2,893 pediatric asthma patients (1,484 in the intervention groups and 1,409 in the control groups). The age of participants spanned from infancy to adolescence, with mean/median ages between 2.04 and 10.89 years across studies.

All trials employed a parallel-group, add-on design. The intervention group in all studies received standard asthma therapy (as per the control group) plus an oral bacterial lysate. The specific product was OM-85 (Broncho-Vaxom) in 27 studies (typical dose: 3.5 mg/day; one study used 7.0 mg/day), and Polyvalent Mechanical Bacterial Lysate (PMBL, Ismigen) in one study (7 mg/day). The control group received standard asthma therapy alone (n = 24) or a placebo (n = 4). Standard therapies varied and included inhaled corticosteroids (ICS) like budesonide or fluticasone, leukotriene receptor antagonists (e.g., montelukast, zafirlukast), long-acting beta-agonists (LABA) in combination, or short-acting bronchodilators.

Treatment duration varied from 14 to 180 days, with the majority of studies (n = 19) employing a 90-day intervention period. Follow-up duration for outcome assessment ranged from the end of treatment up to 12 months post-intervention.

The reported outcome measures were comprehensive, encompassing clinical efficacy (wheezing/exacerbation frequency, time to symptom improvement, respiratory infection rate, overall treatment efficacy rate), lung function (FEV1, FVC, PEF), immunological parameters (T-cell subsets, cytokines, immunoglobulins), inflammatory biomarkers (FeNO, ECP, EOS count), and adverse events. The specific outcomes reported by each study are summarized in Table 1.

**TABLE 1:** Characteristics of included randomized controlled trials.

Study (year)	Country	Sample size (T/C)	Age, years (T/C)	Intervention (T)	Control (C)	Duration (days)	Reported outcomes*
Lin et al. (2024)	China	40/40	10.02 ± 1.01/9.98 ± 0.98	Control therapy + OM-85 (3.5 mg/d)	Montelukast + Budesonide suspension inhalation	90	①,②,③,④,⑧,⑨,⑩
Lu et al. (2023)	China	42/42	8.39 ± 1.28/8.35 ± 1.24	Control therapy + OM-85 (3.5 mg/d)	Budesonide suspension + Montelukast	90	④,⑤,⑥,⑨,⑩
Lin et al. (2023)	China	100/100	7.92 ± 1.53/7.95 ± 1.44	Control therapy + OM-85 (3.5 mg/d)	Budesonide/Formoterol (80 μg + 4.5 μg)	90	④,⑥,⑨,⑩
Shen et al. (2023)	China	53/53	8.82 ± 2.14/8.25 ± 2.05	Control therapy + OM-85 (3.5 mg/d)	Budesonide aerosol	30	④,⑤,⑨,⑩
Luo et al. (2023)	China	45/45	3.49 ± 1.10/3.57 ± 1.05	Control therapy + OM-85 (3.5 mg/d)	Fluticasone propionate inhalation aerosol	90	⑥,⑦,⑨,⑩
Qi et al. (2022)	China	55/55	8.55 ± 1.13/8.83 ± 1.26	Control therapy + OM-85 (3.5 mg/d)	Zafirlukast + Budesonide aerosol	14	④,⑤,⑥,⑨
Wang (2022)	China	31/30	3.22 ± 1.12/3.21 ± 1.01	Control therapy + OM-85 (3.5 mg/d)	Budesonide suspension + Montelukast, Salbutamol + Ipratropium	90	②,⑤,⑥,⑨,⑩
Xu and Xin (2022)	China	47/47	3.71 ± 0.32/3.85 ± 0.37	Control therapy + OM-85 (7.0 mg/d)	Beclomethasone dipropionate suspension	21	②,⑥
Zhu (2021)	China	46/45	7.85 ± 0.68/8.03 ± 0.56	Control therapy + OM-85 (3.5 mg/d)	Budesonide suspension	60	④,⑨,⑩
Jin and Li (2021)	China	45/44	7.84 ± 1.57/7.41 ± 1.25	Control therapy + OM-85 (3.5 mg/d)	Salmeterol/Fluticasone	90	④,⑤,⑥,⑨
Zhang (2021)	China	50/40	5.53 ± 1.06/4.17 ± 1.32	Control therapy + OM-85 (3.5 mg/d)	Budesonide nebulization	30	④,⑥
Yang et al. (2020a)	China	60/60	3.5 ± 0.7/3.5 ± 0.5	Control therapy + OM-85 (3.5 mg/d)	Fluticasone propionate aerosol/Budesonide suspension	90	②,⑤,⑩
Yang (2020)	China	42/42	10.18 ± 0.96/10.52 ± 1.03	Control therapy + OM-85 (3.5 mg/d)	Salmeterol/Fluticasone	90	④,⑤,⑥,⑨
Gao et al. (2010)	China	87/86	3.67/3.67	Control therapy + OM-85 (3.5 mg/d)	Montelukast	90	①,②,③,⑤,⑦,⑩
Zhang and Ding (2019)	China	44/44	6.73 ± 0.82/6.45 ± 0.74	Control therapy + OM-85 (3.5 mg/d)	Budesonide nebulization	30	④,⑨
Tang et al. (2017)	China	44/43	7.8 ± 2.0/7.6 ± 1.9	Control therapy + OM-85 (3.5 mg/d)	Beclomethasone dipropionate aerosol	120	④,⑥,⑨
Wu (2019)	China	49/49	7.43 ± 2.62/7.31 ± 2.71	Control therapy + OM-85 (3.5 mg/d)	Budesonide suspension	90	④,⑥,⑨
Liu (2020)	China	44/44	6.62 ± 0.76/6.41 ± 0.63	Control therapy + OM-85 (3.5 mg/d)	Zafirlukast + Budesonide aerosol	60	⑤,⑨,⑩
Zhang et al. (2014)	China	31/33	6.6 ± 2.1/6.7 ± 2.7	Control therapy + OM-85 (3.5 mg/d)	ICS (unspecified)	90	④,⑥
Hu et al. (2011)	China	47/46	7.78 ± 2.29/8.04 ± 1.84	Control therapy + OM-85 (3.5 mg/d)	Fluticasone propionate inhalation aerosol	90	①,②,③,⑥,⑦,⑩
Yang (2017)	China	43/43	5.30 ± 2.06/5.02 ± 1.82	Control therapy + OM-85 (3.5 mg/d)	Budesonide suspension	21	⑤,⑨
Chen et al. (2015)	China	67/54	5.2 ± 2.2/5.4 ± 2.1	Control therapy + OM-85 (3.5 mg/d)	Budesonide suspension	21	⑤,⑥,⑦,⑨,⑩
Yang et al. (2020b)	China	68/68	6.16 ± 2.57/6.59 ± 2.37	Control therapy + OM-85 (3.5 mg/d)	Salmeterol/Fluticasone	90	①,⑤,⑦,⑧,⑩
Lu et al. (2015)	China	24/36	8.9 ± 2.8/8.7 ± 2.7	Control therapy + OM-85 (3.5 mg/d)	ICS (unspecified)	180	①,②,③,⑤,⑥,⑩
Hu and Wen (2022)	China	66/66	10.89 ± 0.31/10.23 ± 0.24	Control therapy + OM-85 (3.5 mg/d)	Fluticasone/Salmeterol	90	①,②,④,⑤,⑦,⑩
Emeryk et al. (2018)	Poland	74/76	9.3/9.8	Placebo + PMBL (7 mg/d)	Placebo	90	①,②,③,⑩
Han et al. (2016)	China	74/62	2.3 ± 0.6/2.2 ± 0.4	ICS + OM-85 (3.5 mg/d)	ICS (unspecified)	90	①,②,⑥
Razi et al. (2010)	Turkey	35/40	26 months (16–37)/24.5 months (14–45)	Placebo + OM-85 (3.5 mg/d)	Placebo	90	①,②,③,⑩

T, Treatment group; C, Control group.

Outcome codes: ① Wheezing frequency; ② Time to symptom improvement (days); ③ Respiratory tract infection frequency; ④ Lung function (FEV1, FVC, PEF); ⑤ T-lymphocyte subsets (CD3^+^, CD4^+^, CD4+/CD8+); ⑥ Serum cytokines/ECP/EOS levels; ⑦ Salivary sIgA, serum IgM, IgG, IgE; ⑧ FeNO level; ⑨ Overall treatment effective rate; ⑩ Adverse events.

### 3.3 Risk of bias assessment

**FIGURE 2:**
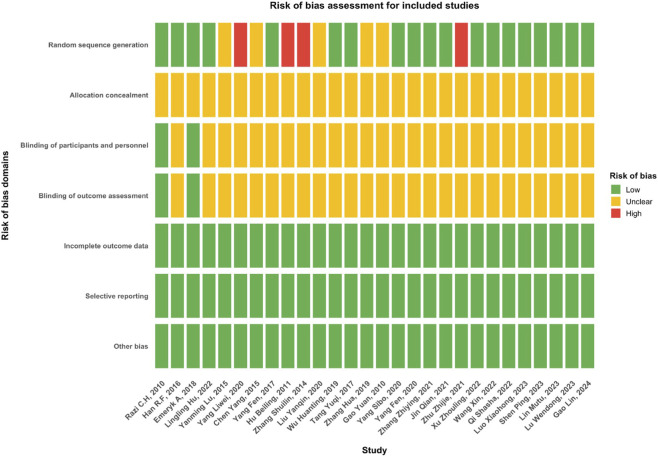
Risk of bias assessment for the 28 included randomized controlled trials using the Cochrane Collaboration's Risk of Bias Tool (RoB 1.0). Each row represents a risk of bias domain, and each column represents an individual study. Color coding indicates the judgment: Green (Low risk) = adequate protection against bias; Yellow (Unclear risk) = insufficient information to permit judgment; Red (High risk) = significant methodological flaws likely to introduce bias. Random sequence generation: 11 studies (39.3%) low risk, 15 (53.6%) unclear, 2 (7.1%) high risk. Allocation concealment: All 28 studies (100%) unclear risk due to no reporting of allocation concealment methods. Blinding of participants and personnel: 26 studies (92.9%) unclear risk, 1 low risk (double-blind), 1 high risk. Blinding of outcome assessment: 26 studies (92.9%) unclear risk, 2 studies (7.1%) low risk. Incomplete outcome data: All 28 studies (100%) low risk. Selective reporting: All 28 studies (100%) low risk. Other bias: All 28 studies (100%) low risk. Overall, the methodological quality was judged as moderate, primarily due to inadequate reporting of allocation concealment and blinding procedures.

The methodological quality of the included studies, assessed using the Cochrane Risk of Bias Tool (RoB 1.0), is summarized in Figure 2. The overall risk of bias was judged as moderate to high, primarily due to inadequate reporting of methodological details.

Random Sequence Generation: Eleven studies (39.3%) were judged as having a low risk of bias, clearly describing appropriate random sequence generation methods (e.g., random number table). For fifteen studies (53.6%), the risk was unclear due to insufficient description (e.g., stated as “randomized” without details). Two studies (7.1%) were judged as high risk.

Allocation Concealment: The risk of bias was unclear for all 28 studies (100%), as none reported the method used to conceal the allocation sequence.

Blinding of Participants and Personnel: Twenty-six studies (92.9%) were rated as having an unclear risk, as blinding of participants and healthcare providers was not mentioned or described. One study (3.6%) was low risk (double-blind), and one was high risk.

Blinding of Outcome Assessment: Similar to performance blinding, the risk was unclear for 26 studies (92.9%). Two studies (7.1%) explicitly reported blinded outcome assessment and were rated low risk.

Incomplete Outcome Data: All 28 studies (100%) were judged as low risk, as they reported complete outcome data or provided acceptable reasons for attrition with balanced groups.

Selective Reporting: All studies (100%) were judged as low risk, as all pre-specified outcomes in the methods were reported in the results sections.

Other Bias: All studies (100%) were rated as low risk for other potential sources of bias.

### 3.4 Results of meta analysis

#### 3.4.1 Clinical efficacy

The clinical efficacy of bacterial lysates was assessed based on the following outcome measures: wheezing/exacerbation frequency, time to symptom improvement, respiratory tract infection (RTI) frequency, and overall treatment efficacy rate.

**FIGURE 3:**
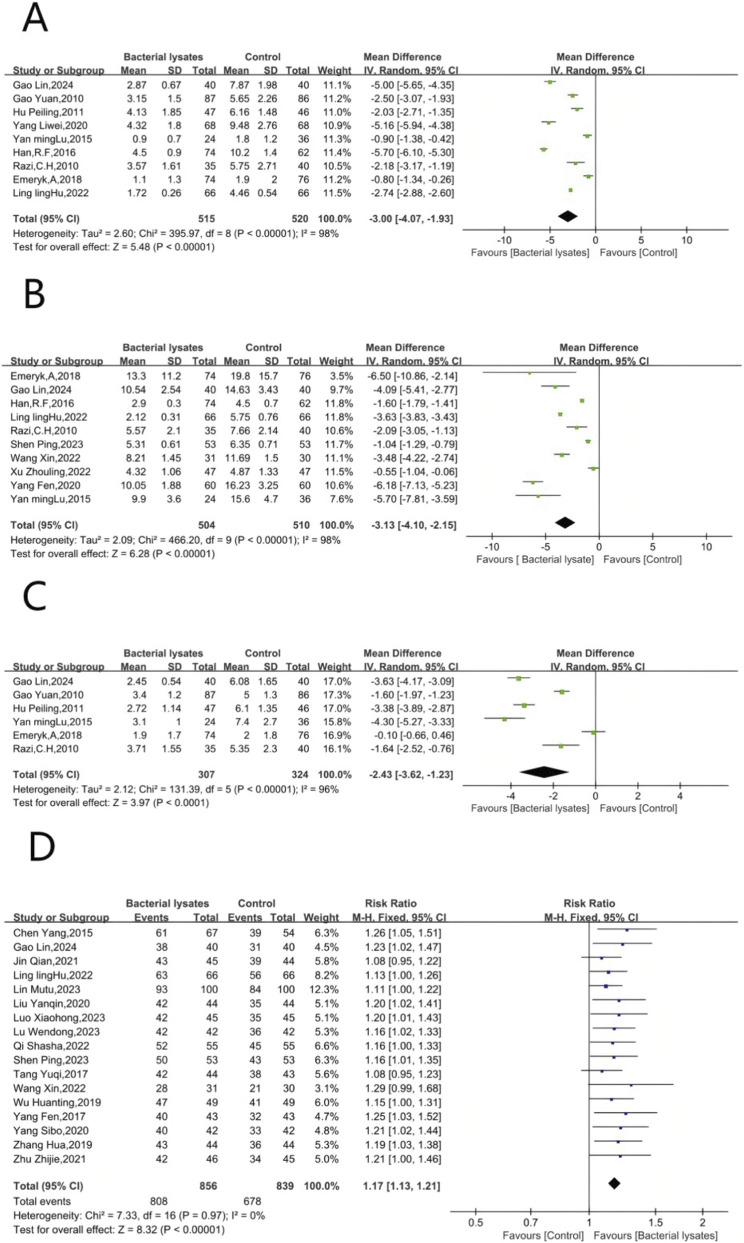
Forest plots of clinical efficacy outcomes comparing bacterial lysates as adjunctive therapy versus control in pediatric asthma. Pooled effect sizes with 95% confidence intervals (CIs) are shown for four primary clinical outcomes. A random-effects model was used for all analyses due to anticipated heterogeneity. Squares represent individual study effect sizes (size proportional to study weight); horizontal lines indicate 95% CIs; diamonds represent pooled estimates. **(A)** Wheezing/exacerbation frequency (9 RCTs, n = 1,035): Adjunctive bacterial lysates significantly reduced the number of wheezing episodes or asthma exacerbations compared with control (Mean Difference [MD] = −3.00, 95% CI: 4.07 to −1.93; Z = 5.50, P < 0.001). Heterogeneity was substantial (I^2^ = 89%, P < 0.001), indicating variability across studies in patient characteristics, follow-up duration, and background therapy. **(B)** Time to symptom improvement (10 RCTs, n = 1,014): Bacterial lysates supplementation shortened the time to improvement of cough and wheezing by approximately 3 days compared with control (MD = −3.13 days, 95% CI: 4.10 to −2.15; Z = 6.27, P < 0.001). High heterogeneity (I^2^ = 85%, P < 0.001) likely reflects differences in definitions of “symptom improvement” across studies. **(C)** Respiratory tract infection (RTI) frequency during follow-up (6 RCTs, n = 636): Bacterial lysates significantly reduced the number of acute RTI episodes (MD = −2.43, 95% CI: 3.62 to −1.23; Z = 3.97, P < 0.001). Heterogeneity was high (I^2^ = 93%, P < 0.001), possibly due to variations in RTI definitions, follow-up duration, and baseline infection susceptibility. **(D)** Overall treatment efficacy rate (17 RCTs, n = 1,695): The proportion of patients classified as “effective” or “markedly effective” based on composite clinical criteria was significantly higher in the bacterial lysates group (Risk Ratio [RR] = 1.17, 95% CI: 1.13 to 1.21; Z = 10.28, P < 0.001). Heterogeneity was modest (I^2^ = 35%, P = 0.07), suggesting reasonable consistency for this outcome. However, Egger’s test suggested potential publication bias (P = 0.03), warranting cautious interpretation.

As shown in Figure 3A, 9 RCTs involving a total of 1,035 participants (515 in the bacterial lysates group and 520 in the control group) reported data on wheezing/exacerbation frequency. The results demonstrated a significant reduction in wheezing/exacerbation frequency in the bacterial lysates group compared with the control group (Mean Difference [MD] = −3.00, 95% Confidence Interval [CI]: 4.07 to −1.93; Z = 5.50, P < 0.001). Heterogeneity was substantial (I^2^ = 89%, P < 0.001), indicating variability across studies in patient characteristics, follow-up duration, and background therapy.

As shown in Figure 3B, 10 RCTs comprising 1,014 participants (504 in the intervention group and 510 in the control group) evaluated the time to symptom improvement. The pooled analysis revealed that bacterial lysates supplementation shortened the time to symptom improvement by approximately 3 days compared with control (MD = −3.13 days, 95% CI: 4.10 to −2.15; Z = 6.27, P < 0.001). High heterogeneity was observed (I^2^ = 85%, P < 0.001), likely reflecting differences in the definition of “symptom improvement” across studies.

As shown in Figure 3C, regarding RTI frequency during follow-up, 6 RCTs were included in the analysis. The meta-analysis suggested that bacterial lysates supplementation significantly reduced the frequency of respiratory tract infections (MD = −2.43, 95% CI: 3.62 to −1.23; Z = 3.97, P < 0.001). Heterogeneity was high (I^2^ = 93%, P < 0.001), possibly due to variations in RTI definitions, follow-up duration, and baseline infection susceptibility.

As shown in Figure 3D, the overall treatment efficacy rate was reported in 17 RCTs, including a total of 1,695 pediatric asthma patients (856 in the bacterial lysates group and 839 in the control group). The results indicated that adjuvant bacterial lysates therapy was associated with an approximately 17% increase in treatment success rate compared to the control group (Risk Ratio [RR] = 1.17, 95% CI: 1.13 to 1.21; Z = 10.28, P < 0.001). Heterogeneity was modest (I^2^ = 35%, P = 0.07), suggesting reasonable consistency for this outcome. However, Egger’s test suggested potential publication bias (P = 0.03), warranting cautious interpretation.

#### 3.4.2 Lung function parameters

As shown in Supplementary Figure S1, meta-analyses of lung function parameters consistently demonstrated statistically significant improvements in the bacterial lysates intervention group compared to controls. For FEV1 (Supplementary Figure S1A), 13 RCTs (n = 2,412 participants) reported a standardized mean difference (SMD) of 0.76 (95% CI: 0.55 to 0.98; Z = 6.96, P < 0.001), indicating a moderate-to-large effect size. However, substantial heterogeneity was observed (I^2^ = 72.0%, P < 0.001), which may reflect variations in spirometry protocols, participant age ranges (from preschool children to adolescents), and differences in concomitant asthma medications (e.g., inhaled corticosteroids vs. leukotriene receptor antagonists). For FVC (Supplementary Figure S1B), a smaller but statistically significant effect was observed (SMD = 0.61, 95% CI: 0.07 to 1.15; Z = 2.19, P = 0.03) across 10 RCTs (n = 1,894), albeit with marked heterogeneity (I^2^ = 94.0%, P < 0.001). For PEF (Supplementary Figure S1C), the largest effect size among lung function outcomes was observed (SMD = 1.12, 95% CI: 0.78 to 1.46; Z = 6.46, P < 0.001), with 11 RCTs (n = 2,135) supporting this finding. The high heterogeneity (I^2^ = 85.0%, P < 0.001) in PEF measurements may stem from disparities in peak flow meter calibration or inconsistent timing of assessments relative to bronchodilator use. Collectively, these results suggest that bacterial lysates may enhance pulmonary function in pediatric asthma, but the variability in effect sizes warrants further investigation into optimal dosing regimens and patient stratification.

#### 3.4.3 Immunological modulation

Bacterial lysates exerted immunomodulatory effects consistent with their proposed mechanism of action. As shown in Supplementary Figure S2, T-lymphocyte subsets showed significant shifts toward Th1 polarization:

CD3+% (Supplementary Figure S2A): Eight RCTs (n = 1,432) reported a significant increase in CD3^+^ T-cell percentage (SMD = 1.78, 95% CI: 1.24 to 2.32; Z = 6.51, P < 0.001). Heterogeneity was high (I^2^ = 88.0%, P < 0.001).

CD4+% (Supplementary Figure S2B): Twelve RCTs (n = 2,132) showed a significant increase in CD4^+^ T-cell percentage (SMD = 1.88, 95% CI: 1.35 to 2.40; Z = 6.96, P < 0.001). Heterogeneity was substantial (I^2^ = 92.0%, P < 0.001).

CD4+/CD8+ ratio (Supplementary Figure S2C): Twelve RCTs (n = 2,132) demonstrated a significant decrease in the CD4+/CD8+ ratio (SMD = −1.73, 95% CI: 2.37 to −1.08; Z = 5.27, P < 0.001), reflecting a rebalancing of cytotoxic and helper T Cell activity. Heterogeneity was high (I^2^ = 94.0%, P < 0.001).

As shown in Supplementary Figure S3, serum cytokines exhibited inconsistent responses:

IL-4 (Supplementary Figure S3A): Fifteen RCTs (n = 2,893) showed a significant reduction in IL-4 levels (SMD = −1.64, 95% CI: 2.35 to −0.94; Z = 4.57, P < 0.001), supporting the hypothesis that bacterial lysates suppress Th2-driven eosinophilic inflammation. Heterogeneity was very high (I^2^ = 96.0%, P < 0.001).

IFN-γ (Supplementary Figure S3B): Fourteen RCTs (n = 2,747) showed no conclusive effect (SMD = 0.71, 95% CI: 0.03 to 1.44; Z = 1.89, P = 0.06). Heterogeneity was very high (I^2^ = 96.0%, P < 0.001), and the result narrowly missed statistical significance.

IL-10 (Supplementary Figure S3C): Eight RCTs (n = 1,271) showed no conclusive effect (SMD = −0.81, 95% CI: 0.03 to 1.65; Z = 1.89, P = 0.06). Heterogeneity was very high (I^2^ = 96.0%, P < 0.001). The inconclusive results for IFN-γ and IL-10 may reflect assay variability, timing of measurement, or insufficient statistical power.

As shown in Supplementary Figure S4, data on salivary sIgA and serum IgM/IgG levels were available from a limited number of eligible studies:

Salivary sIgA (Supplementary Figure S4A): Two RCTs (n = 202) reported significantly higher levels in the bacterial lysates group compared with controls (SMD = 3.64, 95% CI: 2.95 to 4.34; Z = 10.23, P < 0.001). Heterogeneity was low (I^2^ = 0%, P = 0.63). This observation provides partial support for the noted reduction in respiratory tract infections, though the small number of studies limits robustness.

Serum IgM (Supplementary Figure S4B) and IgG (Supplementary Figure S4C): Three RCTs (n = 322) assessed these immunoglobulins; no statistically significant differences were observed between groups for either parameter (IgM: SMD = 0.51, 95% CI: 0.45 to 1.47, P = 0.30; IgG: SMD = 0.29, 95% CI: 0.50 to 1.07, P = 0.47). Heterogeneity was high for both (I^2^ = 86.0%, P < 0.001).

#### 3.4.4 Inflammatory and oxidative stress biomarkers

As shown in Supplementary Figure S5, markers of airway inflammation and oxidative stress were notably reduced in the intervention group:

Eosinophil cationic protein (ECP, Supplementary Figure S5A): 4 RCTs (n = 798) demonstrated a large effect size (SMD = −3.63, 95% CI: 4.67 to −2.60; Z = 6.90, P < 0.001). Heterogeneity was high (I^2^ = 87.0%, P < 0.001). Results are shown after excluding an outlier study with divergent methodology.

Peripheral eosinophil count (EOS, Supplementary Figure S5B): 2 RCTs (n = 113) showed a significant reduction (SMD = −3.71, 95% CI: 4.16 to −3.26; Z = 16.20, P < 0.001). Heterogeneity was low (I^2^ = 0%, P = 0.41), supporting the clinical observation of reduced exacerbation frequency.

Fractional exhaled nitric oxide (FeNO, Supplementary Figure S5C): 2 RCTs (n = 113) showed a significant decline (SMD = −3.36, 95% CI: 4.43 to −2.30; Z = 6.23, P < 0.001). Heterogeneity was substantial (I^2^ = 82.0%, P = 0.02), suggesting variability in FeNO measurement techniques or timing relative to bronchodilator administration.

These findings collectively indicate that bacterial lysates may mitigate eosinophilic inflammation and oxidative stress in pediatric asthma, potentially disrupting the “infection-inflammation-exacerbation” cycle.

#### 3.4.5 Safety profile

**FIGURE 4:**
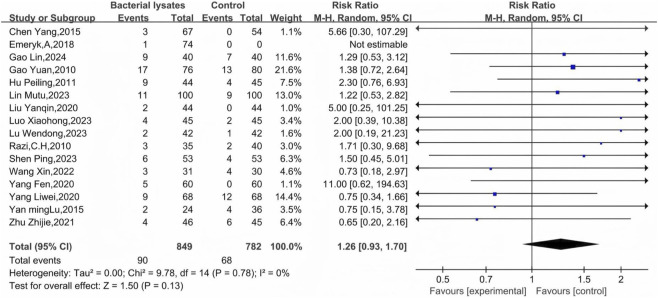
Forest plot of adverse events comparing bacterial lysates as adjunctive therapy versus control in pediatric asthma. Pooled analysis of adverse event incidence from 16 RCTs (n = 2,893). The intervention group did not show a statistically significant increase in overall adverse events compared with the control group (Risk Ratio [RR] = 1.26, 95% CI: 0.93 to 1.70; Z = 1.51, P = 0.13). Heterogeneity was low (I^2^ = 23%, P = 0.20), indicating consistent safety findings across studies. The most commonly reported adverse events were mild and transient, including gastrointestinal symptoms (abdominal pain, nausea, vomiting, diarrhea) and skin rash. No serious adverse events (e.g., anaphylaxis, hepatic dysfunction) were attributed to bacterial lysates in any included study.

As shown in Figure 4, safety outcomes were evaluated in 16 RCTs (n = 2,893 participants). There was no significant increase in overall adverse events in the intervention group (RR = 1.26, 95% CI: 0.93 to 1.70; Z = 1.51, P = 0.13). Heterogeneity was low (I^2^ = 23%, P = 0.20), indicating consistent safety findings across studies. The most commonly reported events were mild and transient, including gastrointestinal symptoms (abdominal pain, nausea, vomiting, diarrhea) and skin rash. No serious adverse events (e.g., anaphylaxis, hepatic dysfunction) were attributed to bacterial lysates in any included study. However, the absence of long-term safety data (>12 months) highlights a critical gap in current evidence.

#### 3.4.6 Publication bias

As shown in .(Supplementary Figure S6–S13), publication bias was assessed through visual inspection of funnel plots for the primary outcomes and incidence of adverse events. The analysis indicated no significant publication bias for adverse events (Supplementary Figure S13), wheezing/exacerbation frequency (Supplementary Figure S6), time to symptom improvement (Supplementary Figure S7), RTI frequency (Supplementary Figure S8), or FEV1 (Supplementary Figure S10). However, discernible asymmetry was observed in funnel plots for PEF (Supplementary Figure S12) and overall treatment efficacy rate (Supplementary Figure S9), suggesting possible publication bias for these outcomes. Egger’s test was statistically significant for overall treatment efficacy rate (P = 0.03) and PEF (P = 0.04). Despite the growing body of research on bacterial lysates in recent years, their underlying mechanisms remain incompletely understood. Future studies should focus on conducting high-quality investigations to further elucidate these mechanisms and strengthen the evidence base.

#### 3.4.7 Heterogeneity

The overall treatment efficacy rate, peripheral eosinophil count, and incidence of adverse events associated with bacterial lysates treatment showed low heterogeneity (I^2^ < 50%), whereas substantial heterogeneity (I^2^ > 50%) was observed across all other outcome variables. This heterogeneity may be attributable to a variety of factors, including differences in patient demographic and clinical characteristics, variations in background therapy, timing of symptom onset, and treatment interventions prior to enrollment. Given the limited number of available studies on bacterial lysates, subgroup or sensitivity analyses to identify sources of heterogeneity were only performed for two key clinical outcomes with notably high heterogeneity (wheezing/exacerbation frequency and time to symptom improvement).

#### 3.4.8 Subgroup and sensitivity analyses

**FIGURE 5:**
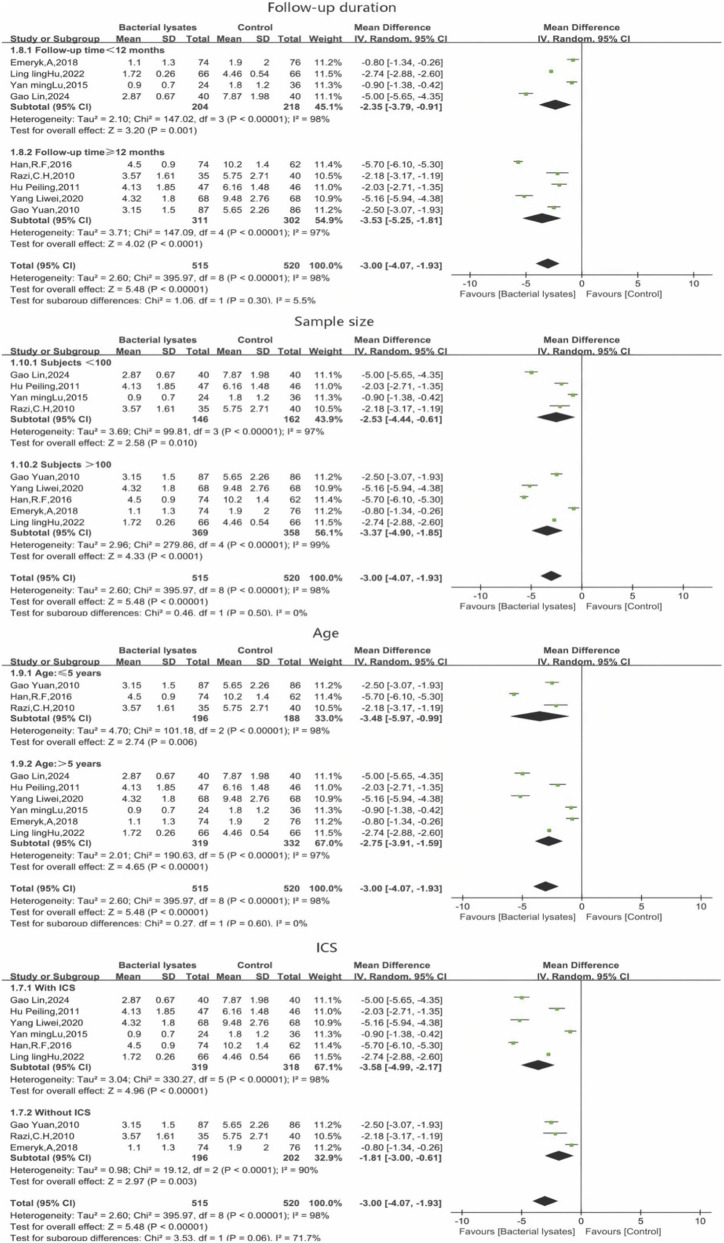
Subgroup analysis of wheezing/exacerbation frequency. Subgroup analyses were performed to explore potential sources of heterogeneity for the outcome of wheezing/exacerbation frequency. Subgroups were defined *a priori* based on concomitant inhaled corticosteroid (ICS) use, follow-up duration, participant age, and study sample size. Concomitant ICS use (8 RCTs): The reduction in wheezing frequency was larger in studies where the control group received ICS therapy (MD = −3.58, 95% CI: 4.99 to −2.17; 5 RCTs) compared to studies without ICS or with placebo (MD = −1.81, 95% CI: 3.00 to −0.61; 3 RCTs). The test for subgroup differences approached statistical significance (Chi^2^ = 3.53, P = 0.06, I^2^ = 71.7%), suggesting that background ICS therapy may be a potential effect modifier. Follow-up duration (9 RCTs): Significant benefits were observed in both short-term (<12 months: MD = −2.35, 95% CI: 3.79 to −0.91; 4 RCTs) and long-term (≥12 months: MD = −3.53, 95% CI: 5.25 to −1.81; 5 RCTs) follow-up. Subgroup differences were not significant (Chi^2^ = 1.06, P = 0.30, I^2^ = 5.5%). Participant age (7 RCTs): Benefits were evident in both younger children (≤5 years: MD = −3.48, 95% CI: 5.97 to −0.99; 3 RCTs) and older children (>5 years: MD = −2.75, 95% CI: 3.91 to −1.59; 4 RCTs). Subgroup differences were not significant (Chi^2^ = 0.27, P = 0.60, I^2^ = 0%). Study sample size (9 RCTs): Benefits were observed in both smaller studies (<100 participants: MD = −2.53, 95% CI: 4.44 to −0.61; 5 RCTs) and larger studies (≥100 participants: MD = −3.37, 95% CI: 4.90 to −1.85; 4 RCTs). Subgroup differences were not significant (Chi^2^ = 0.46, P = 0.50, I^2^ = 0%).

**FIGURE 6:**
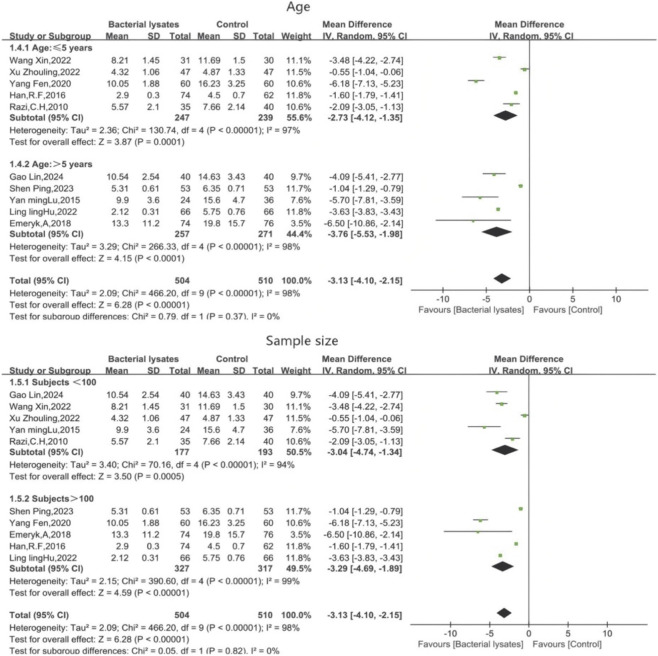
Subgroup analysis of time to symptom improvement. Subgroup analyses for the outcome of time to symptom improvement (days) based on participant age and study sample size. Participant age (8 RCTs): Both younger children (≤5 years: MD = −2.67 days, 95% CI: 4.04 to −1.30; 3 RCTs) and older children (>5 years: MD = −3.78 days, 95% CI: 5.56 to −2.00; 5 RCTs) showed significant reductions in symptom improvement time. Subgroup differences were not significant (Chi^2^ = 0.79, P = 0.37, I^2^ = 0%). Study sample size (10 RCTs): Both smaller studies (<100 participants: MD = −3.04 days, 95% CI: 4.74 to −1.34; 6 RCTs) and larger studies (≥100 participants: MD = −3.29 days, 95% CI: 4.69 to −1.89; 4 RCTs) demonstrated significant benefits. Subgroup differences were not significant (Chi^2^ = 0.05, P = 0.82, I^2^ = 0%).

To explore potential sources of heterogeneity, pre-specified subgroup analyses were conducted for the primary clinical outcomes of wheezing/exacerbation frequency (Figure 5) and time to symptom improvement (Figure 6). Subgroups were defined by: concomitant ICS use (Yes vs. No/Placebo), follow-up duration (<12 months vs. ≥12 months), participant age (≤5 years vs. >5 years), and study sample size (<100 vs. ≥100 participants).

For wheezing/exacerbation frequency (Figure 5):

Concomitant ICS use: Wheezing frequency was significantly reduced regardless of ICS use in the control regimen, but the reduction was larger in studies where the control group received ICS (MD = −3.58, 95% CI: 4.99 to −2.17; 5 RCTs) compared to studies without ICS or on placebo (MD = −1.81, 95% CI: 3.00 to −0.61; 3 RCTs). The test for subgroup differences approached statistical significance (Chi^2^ = 3.53, P = 0.06, I^2^ = 71.7%), suggesting that background ICS therapy may be a potential effect modifier.

Follow-up duration: Significant benefits were observed in both the <12 months (MD = −2.35, 95% CI: 3.79 to −0.91; 4 RCTs) and ≥12 months (MD = −3.53, 95% CI: 5.25 to −1.81; 5 RCTs) subgroups. Subgroup differences were not significant (Chi^2^ = 1.06, P = 0.30, I^2^ = 5.5%).

Participant age: Significant reductions were observed in both the ≤5 years (MD = −3.48, 95% CI: 5.97 to −0.99; 3 RCTs) and >5 years (MD = −2.75, 95% CI: 3.91 to −1.59; 4 RCTs) subgroups. Subgroup differences were not significant (Chi^2^ = 0.27, P = 0.60, I^2^ = 0%).

Sample size: Significant benefits were observed in both the <100 participants (MD = −2.53, 95% CI: 4.44 to −0.61; 5 RCTs) and ≥100 participants (MD = −3.37, 95% CI: 4.90 to −1.85; 4 RCTs) subgroups. Subgroup differences were not significant (Chi^2^ = 0.46, P = 0.50, I^2^ = 0%).

For time to symptom improvement (Figure 6):

Participant age: Time to symptom improvement was significantly shorter in both the ≤5 years (MD = −2.67 days, 95% CI: 4.04 to −1.30; 3 RCTs) and >5 years (MD = −3.78 days, 95% CI: 5.56 to −2.00; 5 RCTs) subgroups. Subgroup differences were not significant (Chi^2^ = 0.79, P = 0.37, I^2^ = 0%).

Sample size: Significant reductions were found in both the <100 participants (MD = −3.04 days, 95% CI: 4.74 to −1.34; 6 RCTs) and ≥100 participants (MD = −3.29 days, 95% CI: 4.69 to −1.89; 4 RCTs) subgroups. Subgroup differences were not significant (Chi^2^ = 0.05, P = 0.82, I^2^ = 0%).

Additionally, sensitivity analysis using the leave-one-out method demonstrated that the pooled effect sizes and heterogeneity for the primary outcomes were not substantially altered by the removal of any single study, indicating overall robustness of the findings. However, as noted above, Egger’s test suggested potential publication bias for the overall treatment efficacy rate and PEF outcomes, warranting caution in their interpretation.

## 4 Discussion

This systematic review and meta-analysis of 28 RCTs, encompassing 2,893 pediatric patients, synthesizes the current evidence on the adjunctive use of oral bacterial lysates in childhood asthma. Our findings suggest that bacterial lysates, primarily OM-85/Broncho-Vaxom, confer statistically significant benefits across a spectrum of outcomes when added to standard asthma therapy. These benefits include a reduction in the frequency of wheezing episodes and respiratory infections, an acceleration of symptom resolution, improvements in lung function parameters, and favorable modulations of key immunological and inflammatory biomarkers, all without a significant increase in adverse events. These results lend support to the proposed immunomodulatory mechanism of action for bacterial lysates and position them as a potential complementary strategy in pediatric asthma management, particularly for children prone to infection-triggered exacerbations.

### 4.1 Integration of clinical and mechanistic evidence

The observed clinical benefits appear coherent with the postulated immunomodulatory properties of bacterial lysates. The significant reduction in wheezing frequency aligns with the conceptual model of disrupting the “infection-inflammation-exacerbation” cycle, a common pattern in pediatric asthma where viral respiratory infections are primary triggers (Jackson and Gern, 2022; Richard et al., 2015). By potentially enhancing innate and mucosal immunity, bacterial lysates may reduce the incidence or severity of these triggering infections, thereby preventing downstream inflammatory cascades that lead to bronchoconstriction and symptoms (Agnieszka et al., 2022; Fabio et al., 2020; Esposito et al., 2019). This is further corroborated by the significant reduction in the frequency of reported respiratory tract infections in our analysis. The improvement in the time to symptom improvement, while statistically significant, should be interpreted with caution due to high heterogeneity, which may reflect differences in how “improvement” was defined and measured across studies.

The pattern of immunological changes observed provides a plausible biological substrate for these clinical effects. The consistent increase in peripheral CD3^+^ and CD4^+^ T-cell percentages, coupled with a decrease in the CD4+/CD8+ ratio, suggests an immunomodulatory shift. While often simplistically framed as a “Th1/Th2 rebalancing”,the reality is likely more nuanced. The significant reduction in serum IL-4, a canonical Th2 cytokine central to allergic inflammation, IgE production, and eosinophil recruitment, is a compelling finding that supports a direct effect on a key pathological pathway in asthma (Gans Melissa and Gavrilova, 2019). This aligns with the known pharmacological action of bacterial lysates like OM-85 (Yaling et al., 2025). This is further strengthened by the observed reductions in eosinophil (EOS) count and eosinophil cationic protein (ECP), markers of eosinophilic airway inflammation (Habib et al., 2022). The lack of statistically significant effects on IFN-γ (a Th1 cytokine) and IL-10 (an anti-inflammatory/regulatory cytokine) is not necessarily contradictory. It may indicate that the primary immunomodulatory action in this context is the dampening of established Th2-driven inflammation rather than a robust polarization toward a Th1 response or a strong induction of regulatory circuits. Alternatively, it may reflect assay variability, timing of measurement, or insufficient statistical power for these specific outcomes.

### 4.2 Interpretation of pulmonary function and biomarker findings

The improvements in lung function (FEV1, FVC, PEF), while statistically significant, were characterized by considerable heterogeneity. This heterogeneity is unsurprising given the wide age range of participants (infants to adolescents), varying degrees of asthma severity and control at baseline, and differences in the background standard therapy (e.g., ICS dose, use of LTRA),all of which are known to influence spirometric outcomes in pediatric asthma (Richard et al., 2024). The physiological meaning of these improvements, particularly the larger standardized effect size for PEF compared to FEV1, warrants consideration. PEF is an effort-dependent measure that can be more variable and is sensitive to acute bronchodilator effects, whereas FEV1 is considered a more robust and reproducible indicator of airflow obstruction (Arnold Donald et al., 2020). The concurrent reduction in FeNO, a well-established non-invasive biomarker of type 2 (eosinophilic) airway inflammation in asthma (Kay et al., 2023), provides a link between the immunological changes and the physiological improvement, suggesting that better asthma control and lung function may stem, at least in part, from a reduction in underlying eosinophilic inflammation (Chun et al., 2025).

### 4.3 Safety and tolerability in the pediatric population

The safety profile emerging from this analysis is reassuring and consistent with the extensive post-marketing experience with OM-85,which reports a favorable long-term safety record (Cao et al., 2021; Joanna et al., 2026). The absence of a statistically significant increase in the overall risk of adverse events, and the lack of reported serious adverse events directly attributable to the bacterial lysates, are critical findings for a preventive adjunctive therapy intended for use in children,a population in whom safety considerations are paramount (José et al., 2025; Kristina and Ralph Edwards, 2014). The reported events were predominantly mild, transient gastrointestinal symptoms, which are common to many oral medications and seldom lead to treatment discontinuation, which aligns with the known and characteristic adverse effect profile of OM-85 (Esposito et al., 2019; Solèr et al., 2006; Ferah and Kutukculer, 2003) and are common to many oral medications, seldom leading to treatment discontinuation. This favorable safety profile is a key strength when considering the long-term or repeated seasonal use that might be envisaged for such an immunomodulator.

### 4.4 Critical appraisal of limitations and sources of heterogeneity

While the findings of this meta-analysis are encouraging, they must be interpreted within the context of substantial methodological limitations and significant heterogeneity, which collectively temper the strength of the conclusions and rigorously define the scope for future research. The overall methodological quality of the included trials was moderate, with a predominant and concerning risk of “unclear” bias pertaining to allocation concealment and blinding—key safeguards against performance and detection bias, especially for subjective clinician-assessed outcomes such as treatment effectiveness (Higgins Julian et al., 2011). Furthermore, the marked geographical concentration of evidence (24 of 28 studies from China) may limit the generalizability of findings to other healthcare systems and ethnic populations, while statistical and visual indicators suggest potential publication bias for the efficacy rate outcome, risking an overestimation of this treatment effect.

A central challenge in synthesizing the evidence is the pervasive and substantial statistical heterogeneity (I^2^ > 70% for most outcomes), which signifies important clinical and methodological diversity across studies. This heterogeneity stems from multiple sources: the inclusion of a broad pediatric age range (preschoolers to adolescents) with differing asthma pathophysiologies and potentially distinct endotypes (Picheswara Rao and Krishna Bikki, 2025; Anuradha et al., 2020); variability in the intervention’s dosage, duration, and administration schedule; the heterogeneous composition of “standard therapy” in control groups, ranging from placebo to various regimens of inhaled corticosteroids and/or leukotriene receptor antagonists; and a lack of standardization in defining and measuring outcomes such as exacerbations, infections, and even “clinical effectiveness”,which is a recognized challenge in pediatric asthma trials (Ekaterina et al., 2022; Sinha Ian et al., 2012; Szefler, 2001). The “overall treatment efficacy rate” warrants specific scrutiny as a composite endpoint. Although a statistically significant benefit was observed (RR = 1.17), this measure is typically a dichotomous, investigator-assessed judgment based on variable combinations of symptoms, signs, and exacerbation frequency, with no standardized definition across studies. This lack of uniformity introduces significant measurement bias and heterogeneity, which likely explains the observed publication bias for this outcome. As such, the clinical relevance of this pooled estimate remains uncertain. This finding strongly advocates for the use of objective, validated core outcome sets in pediatric asthma trials, such as standardized symptom scores (e.g.,,the Asthma Control Questionnaire [ACQ] for older children and adolescents, or the Childhood Asthma Control Test [C-ACT] for younger children) or protocol-defined exacerbations, to enhance evidence reliability and clinical applicability. Among the pre-specified subgroup analyses, the potential interaction between bacterial lysates and background inhaled corticosteroid (ICS) therapy warrants closer examination. The reduction in wheezing frequency appeared more pronounced in children already receiving ICS (MD = −3.58) compared to those without ICS or on placebo (MD = −1.81; subgroup difference P = 0.06). Although exploratory, this finding raises a biologically plausible hypothesis: in patients under ICS treatment, partial suppression of Th2-driven inflammation may allow the immunomodulatory and anti-infective effects of bacterial lysates to more effectively prevent viral-triggered breakthrough exacerbations. Conversely, in non-steroid individuals, the unmodulated Th2 milieu might attenuate the bacterial lysates’ subtler immunomodulatory signal. If confirmed, this would suggest bacterial lysates could be optimally targeted as adjuvant therapy for a specific phenotype—children with ICS-treated asthma who remain prone to recurrent, infection-induced exacerbations. This refined hypothesis shifts the focus from a broad pediatric asthma population to a higher-risk subset on controller therapy, and should be prospectively evaluated in future trials. However, these subgroup analyses were unable to fully account for the high degree of statistical heterogeneity observed within each subgroup. This persistent heterogeneity indicates that beyond broad treatment context, other unmeasured or unreported factors—such as variations in asthma endotypes (e.g., Th2-high vs. Th2-low phenotype) (Francesco and Schaub, 2023; Kuruvilla Merin et al., 2018), the timing and duration of OBLs administration relative to the respiratory infection season, genetic background, or adherence to concomitant medications—likely contribute to the variable treatment responses. The inability of our analyses to resolve this heterogeneity underscores the complexity of pediatric asthma and the non-uniform patient populations enrolled in the current trials.

Additional limitations include the deliberate exclusion of smaller studies (n < 60), which may have introduced selection bias, and the restriction to English and Chinese publications, which may have omitted relevant data. Finally, the predominantly short-to medium-term follow-up of the included trials precludes any assessment of the long-term sustainability of clinical effects, potential disease-modifying actions, or the safety profile of bacterial lysates used chronically in developing immune systems.

### 4.5 Implications for clinical practice and future research

Given the constellation of limitations outlined above, it is premature to derive definitive clinical practice guidelines from the current evidence base. Nevertheless, the consistent signal of benefit across multiple clinically relevant outcome domains, coupled with a favorable safety profile, positions bacterial lysates as a promising and biologically plausible therapeutic strategy. They may warrant consideration, within a shared decision-making framework, for a targeted subset of the pediatric asthma population—particularly those experiencing frequent viral-triggered exacerbations where enhancing innate respiratory defence is a strategic therapeutic goal (Holt and Sly, 2012). The potential interaction with background ICS therapy, as suggested by our subgroup analysis, implies that the additive benefit may vary depending on the existing controller regimen. Clinicians should weigh this option against the cost of treatment and individual patient circumstances.

To conclusively establish the role and value of bacterial lysates in asthma management, future research must be guided by a precision medicine approach that addresses the identified gaps (Deng et al., 2022; Lou et al., 2025a). Based on the findings and limitations of this systematic review, we propose the following specific questions to guide future investigations: (1) Can the observed benefits of OBLs be confirmed in large, multicenter, double-blind, placebo-controlled RCTs with rigorous methodology (e.g., adequate allocation concealment, blinding of outcome assessors)? Current evidence is limited by moderate methodological quality and potential bias. (2) Which specific pediatric asthma phenotypes or endotypes (e.g., children with ICS-treated asthma who remain prone to recurrent infection-induced exacerbations, Th2-high vs. Th2-low endotypes) derive the greatest benefit from adjunctive OBLs? The exploratory subgroup analysis suggesting effect modification by background ICS therapy (P = 0.06) warrants prospective confirmation. (3) What are the long-term safety and efficacy outcomes of OBLs when used chronically or repeatedly over periods exceeding 12 months? Current evidence is limited to short- and medium-term follow-up. (4) What is the optimal dosing regimen, treatment duration, and timing of OBLs administration (e.g., seasonal prophylaxis vs. continuous therapy) to maximize clinical benefit? Heterogeneity in intervention protocols across included studies precludes definitive recommendations. (5) Can the immunological mechanisms of OBLs be further elucidated using standardized biomarker panels (e.g., Th1/Th2/Th17 cytokines, regulatory T-cell markers, innate immune parameters) measured at multiple time points? Current cytokine data showed high heterogeneity and inconclusive results for IFN-γ and IL-10. (6) What is the cost-effectiveness of OBLs as an adjunctive therapy in different healthcare systems, considering potential reductions in exacerbations, hospitalizations, and antibiotic use?

The highest priority should be accorded to large, pragmatic, multicenter randomized controlled trials conducted in diverse populations. These trials must be prospectively designed to test whether specific subgroups, such as children on background ICS therapy or those with a history of frequent viral-triggered exacerbations and specific immune profiles (e.g., Th2-high) (Gans Melissa and Gavrilova, 2019), derive differential benefit. They must employ rigorous methodology with explicit reporting of randomization, allocation concealment, and blinding, and utilize well-defined, optimized standard therapy as the active comparator. The adoption of a core outcome set for pediatric asthma research is essential to ensure consistency in defining and measuring exacerbations, symptom control, and infections (Ekaterina et al., 2022; Lou et al., 2025b; Lou et al., 2025c). Furthermore, long-term extension studies (≥2 years) are imperative to evaluate sustained efficacy, disease-modifying potential, and long-term safety. Finally, comprehensive health economic analyses are needed to determine the cost-effectiveness of this adjunctive therapy within different healthcare contexts.

## 5 Conclusion

Adjunctive oral bacterial lysates therapy is associated with significant clinical improvements in pediatric asthma, including reduced wheezing frequency (MD = −3.00), shorter symptom recovery time (MD = −3.13 days), and higher treatment response rates (RR = 1.27). Benefits extend to lung function and immunomodulation, notably increased CD3+/CD4+ T cells and reduced IL-4 and eosinophilic markers, without a significant increase in adverse events. However, these findings are constrained by moderate methodological quality, substantial unexplained heterogeneity—partially related to background ICS use—and potential publication bias. Therefore, while OBLs represent a promising adjunct, especially in infection-prone children, their routine use cannot yet be recommended. Nonetheless, it holds promise as a safe, theory-supported adjunct worthy of consideration for children whose asthma remains susceptible to recurrent infection-induced exacerbations despite standard care. Definitive conclusions await larger, rigorously designed, long-term trials that adopt standardized outcomes and account for phenotypic predictors of response.

## Data Availability

The original contributions presented in the study are included in the article/Supplementary Material, further inquiries can be directed to the corresponding author.

## Supplementary material

SUPPLEMENTARY FIGURE S1 Forest plots of lung function parameters: FEV1, FVC, and PEF. Meta-analyses of lung function outcomes measured as standardized mean differences (SMD) with 95% CIs. A random-effects model was used for all analyses due to substantial heterogeneity. Forced Expiratory Volume in 1 s (FEV1, 13 RCTs, n = 2,412): Bacterial lysates significantly improved FEV1 compared with control (SMD = 0.76, 95% CI: 0.55 to 0.98; Z = 6.96, P < 0.001). Heterogeneity was high (I^2^ = 72.0%, P < 0.001), indicating moderate-to-large effect size but variability across studies. Forced Vital Capacity (FVC, 10 RCTs, n = 1,894): A smaller but statistically significant improvement was observed in FVC (SMD = 0.61, 95% CI: 0.07 to 1.15; Z = 2.19, P = 0.03). Heterogeneity was very high (I^2^ = 94.0%, P < 0.001), suggesting caution in interpretation. Peak Expiratory Flow (PEF, 11 RCTs, n = 2,135): The largest effect size among lung function parameters was observed for PEF (SMD = 1.12, 95% CI: 0.78 to 1.46; Z = 6.46, P < 0.001). Heterogeneity was high (I^2^ = 85.0%, P < 0.001), likely due to variations in peak flow meter calibration and timing of assessments.

SUPPLEMENTARY FIGURE S2 Forest plots of T-lymphocyte subsets: CD3+%, CD4+%, and CD4+/CD8+ ratio.Pooled analyses of T-lymphocyte subsets measured as standardized mean differences (SMD) with 95% CIs. All outcomes showed significant immunomodulatory effects favoring the bacterial lysates group. CD3+% (8 RCTs, n = 1,432): Bacterial lysates significantly increased CD3^+^ T-cell percentage (SMD = 1.78, 95% CI: 1.24 to 2.32; Z = 6.51, P < 0.001). Heterogeneity was high (I^2^ = 88.0%, P < 0.001). CD4+% (12 RCTs, n = 2,132): A significant increase in CD4^+^ T-cell percentage was observed (SMD = 1.88, 95% CI: 1.35 to 2.40; Z = 6.96, P < 0.001). Heterogeneity was substantial (I^2^ = 92.0%, P < 0.001). CD4+/CD8+ ratio (12 RCTs, n = 2,132): The CD4+/CD8+ ratio significantly decreased in the bacterial lysates group compared with control (SMD = −1.73, 95% CI: 2.37 to −1.08; Z = 5.27, P < 0.001), indicating a rebalancing of helper and cytotoxic T-cell activity. Heterogeneity was high (I^2^ = 94.0%, P < 0.001).

SUPPLEMENTARY FIGURE S3 Forest plots of serum cytokines: IL-4, IFN-γ, and IL-10. Pooled analyses of serum cytokine levels measured as standardized mean differences (SMD) with 95% CIs. A random-effects model was used for all analyses. Interleukin-4 (IL-4, 15 RCTs, n = 2,893): Bacterial lysates significantly reduced IL-4 levels, a canonical Th2 cytokine central to allergic inflammation (SMD = −1.64, 95% CI: 2.35 to −0.94; Z = 4.57, P < 0.001). Heterogeneity was very high (I^2^ = 96.0%, P < 0.001). Interferon-γ (IFN-γ, 14 RCTs, n = 2,747): No statistically significant effect was observed for IFN-γ (SMD = 0.71, 95% CI: 0.03 to 1.44; Z = 1.89, P = 0.06). Heterogeneity was very high (I^2^ = 96.0%, P < 0.001), and the result narrowly missed statistical significance. Interleukin-10 (IL-10, 8 RCTs, n = 1,271): No statistically significant effect was observed for IL-10 (SMD = −0.81, 95% CI: 0.03 to 1.65; Z = 1.89, P = 0.06). Heterogeneity was very high (I^2^ = 96.0%, P < 0.001). The inconclusive results for IFN-γ and IL-10 may reflect assay variability, timing of measurement, or insufficient statistical power.

SUPPLEMENTARY FIGURE S4 Forest plots of immunoglobulin levels: salivary sIgA, serum IgM, and serum IgG. Pooled analyses of immunoglobulin levels measured as standardized mean differences (SMD) with 95% CIs. Salivary secretory immunoglobulin A (sIgA, 2 RCTs, n = 202): Bacterial lysates significantly increased salivary sIgA levels compared with control (SMD = 3.64, 95% CI: 2.95 to 4.34; Z = 10.23, P < 0.001). Heterogeneity was low (I^2^ = 0%, P = 0.63). This finding provides partial support for the observed reduction in respiratory tract infections, though the small number of studies limits robustness. Serum immunoglobulin M (IgM, 3 RCTs, n = 322): No statistically significant difference was observed between groups (SMD = 0.51, 95% CI: 0.45 to 1.47; Z = 1.04, P = 0.30). Heterogeneity was high (I^2^ = 86.0%, P < 0.001). Serum immunoglobulin G (IgG, 3 RCTs, n = 322): No statistically significant difference was observed between groups (SMD = 0.29, 95% CI: 0.50 to 1.07; Z = 0.72, P = 0.47). Heterogeneity was high (I^2^ = 86.0%, P < 0.001).

SUPPLEMENTARY FIGURE S5 Forest plots of inflammatory and oxidative stress biomarkers: ECP, EOS, and FeNO. Pooled analyses of biomarkers of eosinophilic inflammation and airway inflammation measured as standardized mean differences (SMD) with 95% CIs. Eosinophil cationic protein (ECP, 4 RCTs, n = 798): Bacterial lysates significantly reduced serum ECP levels, a marker of eosinophil activation (SMD = −3.63, 95% CI: 4.67 to −2.60; Z = 6.90, P < 0.001). Heterogeneity was high (I^2^ = 87.0%, P < 0.001). Results are shown after excluding an outlier study with divergent methodology. Peripheral eosinophil count (EOS, 2 RCTs, n = 113): A significant reduction in peripheral eosinophil count was observed (SMD = −3.71, 95% CI: 4.16 to −3.26; Z = 16.20, P < 0.001). Heterogeneity was low (I^2^ = 0%, P = 0.41), supporting the clinical observation of reduced exacerbation frequency. Fractional exhaled nitric oxide (FeNO, 2 RCTs, n = 113): Bacterial lysates significantly reduced FeNO levels, a non-invasive marker of type 2 airway inflammation (SMD = −3.36, 95% CI: 4.43 to −2.30; Z = 6.23, P < 0.001). Heterogeneity was substantial (I^2^ = 82.0%, P = 0.02), suggesting variability in FeNO measurement techniques or timing relative to bronchodilator administration.

SUPPLEMENTARY FIGURE S6 Wheezing/exacerbation frequency (9 RCTs): Funnel plot appeared roughly symmetric, with no strong evidence of publication bias (Egger’s test P = 0.21).

SUPPLEMENTARY FIGURE S7 Time to symptom improvement (10 RCTs): Funnel plot showed mild asymmetry, but Egger’s test was not statistically significant (P = 0.09).

SUPPLEMENTARY FIGURE S8 RTI frequency (6 RCTs): Limited number of studies precluded formal testing; visual inspection suggested no clear asymmetry.

SUPPLEMENTARY FIGURE S9 Overall treatment efficacy rate (17 RCTs): Funnel plot showed discernible asymmetry, and Egger’s test was statistically significant (P = 0.03), suggesting potential publication bias. This finding warrants caution in interpreting the pooled RR of 1.17 for this outcome.

SUPPLEMENTARY FIGURE S10 FEV1 (13 RCTs): Funnel plot appeared generally symmetric (Egger’s test P = 0.14).

SUPPLEMENTARY FIGURE S11 FVC (10 RCTs): Funnel plot showed mild asymmetry (Egger’s test P = 0.08).

SUPPLEMENTARY FIGURE S12 PEF (11 RCTs): Funnel plot demonstrated noticeable asymmetry, suggesting possible publication bias (Egger’s test P = 0.04).

SUPPLEMENTARY FIGURE S13 Adverse events (16 RCTs): Funnel plot was symmetric (Egger’s test P = 0.31), indicating no significant publication bias for safety outcomes.

## References

[B1] Adnan C. Marinho S. Simpson A. (2012). Gene-environment interactions in the development of asthma and atopy. Expert Rev. Respir. Med. 6 (3). 10.1586/ers.12.24 22788944

[B2] Adriana R. Chorostowska-Wynimko J. (2008). Bacterial immunostimulants--mechanism of action and clinical application in respiratory diseases. Pneumonol. Alergol. Pol. 76 (5). 19003766

[B3] Agnieszka K. Klosinska M. Janeczek K. (2022). Promising immunomodulatory effects of bacterial lysates in allergic diseases. Front. Immunol. 13 (0). 10.3389/fimmu.2022.907149 PMC9257936 35812388

[B4] Anuradha R. Camiolo M. Fitzpatrick A. (2020). Are we meeting the promise of endotypes and precision medicine in asthma? Physiol. Rev. 100 (3). 10.1152/physrev.00023.2019 PMC7474260 31917651

[B5] Arnold Donald H. Lindsell C. J. Wu G. (2020). Peak expiratory flow and forced expiratory volume in 1 second percent predicted values are not interchangeable pediatric asthma exacerbation severity measures. Ann. Am. Thorac. Soc. 17 (5). 10.1513/AnnalsATS.201909-684RL PMC7193809 31990222

[B6] Asher I. Pearce N. (2014). Global burden of asthma among children. Int. J. Tuberc. Lung Dis. 18 (11), 1269–1278. 10.5588/ijtld.14.0170 25299857

[B7] Barnes P. J. (2008). Immunology of asthma and chronic obstructive pulmonary disease. Nat. Rev. Immunol. 8 (3), 183–192. 10.1038/nri2254 18274560

[B8] Bateman E. D. Hurd S. S. Barnes P. J. Bousquet J. Drazen J. M. FitzGerald J. M. (2008). Global strategy for asthma management and prevention: GINA executive summary. Eur. Respir. J. 51 (2), 143–178. 10.1183/09031936.00138707 18166595

[B9] Bloomfield Sally F. Rook G.A. Scott E. A. Shanahan F. Stanwell-Smith R. Turner P. (2016). Time to abandon the hygiene hypothesis: new perspectives on allergic disease, the human microbiome, infectious disease prevention and the role of targeted hygiene. Perspect. Public Health 136 (4), 213–224. 10.1177/1757913916650225 27354505 PMC4966430

[B10] Busse W. W. Lemanske R. F. Jr Gern J. E. (2010). Role of viral respiratory infections in asthma and asthma exacerbations. Lancet 376 (9743), 826–834. 10.1016/S0140-6736(10)61380-3 20816549 PMC2972660

[B11] Cao C. Wang J. Li Y. Li Y. Ma L. Abdelrahim M. E. A. (2021). Efficacy and safety of OM-85 in paediatric recurrent respiratory tract infections which could have a possible protective effect on COVID-19 pandemic: a meta-analysis. Int. J. Clin. Pract. 75 (5). 10.1111/ijcp.13981 PMC7883224 33405321

[B12] Castro-Rodriguez J. A. Kedir N. T. Forno E. (2024). A critical analysis of the effect of OM-85 for the prevention of recurrent respiratory tract infections or wheezing/asthma from systematic reviews with meta-analysis. Pediatr. Allergy Immunol. 35 (7). 10.1111/pai.14186 PMC11296687 39016384

[B13] Chen Y. Zhu F. Li Q. (2015). Clinical study on effect of broncho-vaxom combined with budesonide atomization inhalation on immune function of children with bronchial asthma. Chin. J. Clin. Pharmacol. 31 (6).

[B14] Chun K. W. Mary S. M.Ip Tam T. C. C. (2025). Correlation of FE(NO) with spirometric measurements and blood eosinophil level in patients with severe asthma. Clin. Respir. J. 19 (7). 10.1111/crj.70094 PMC12287615 40702961

[B15] Deng Y. Xu X. Meng F. Lou J. Liao Y. Li Q. (2022). PRP8-Induced CircMaml2 facilitates the healing of the intestinal mucosa *via* recruiting PTBP1 and regulating Sec62. Cells 11 (21), 3460. 10.3390/cells11213460 36359856 PMC9654005

[B16] Ekaterina K. Rattu A. Brightling C. (2022). Development of core outcome measures sets for paediatric and adult severe asthma (COMSA). Eur. Respir. J. 61 (4). 10.1183/13993003.00606-2022 PMC10069873 36229046

[B17] Elpida C. M. Panteli L. S. Moisidis J. A. (2025). Oral bacterial lysate OM-85 prevents respiratory tract infections in asthma: the OMRIA RWE study. J. Asthma Allergy 18 (0). 10.2147/JAA.S517194 PMC12143292 40487798

[B18] Emeryk A. Bartkowiak-Emeryk M. Raus Z. Braido F. Ferlazzo G. Melioli G. (2018). Mechanical bacterial lysate administration prevents exacerbation in allergic asthmatic children—The EOLIA study. Pediatr. Allergy Immunol. 29 (4), 394–401. 10.1111/pai.12894 29575037

[B19] Esposito S. Bianchini S. Bosis S. Tagliabue C. Coro I. Argentiero A. (2019). A randomized, placebo-controlled, double-blinded, single-centre, phase IV trial to assess the efficacy and safety of OM-85 in children suffering from recurrent respiratory tract infections. J. Transl. Med. 17 (1), 284. 10.1186/s12967-019-2040-y 31443716 PMC6708164

[B20] Fabio C. Lombardi E. Rossi O. (2020). Epithelial dysfunction, respiratory infections and asthma: the importance of immunomodulation. A focus on OM-85. Expert Rev. Respir. Med. 14 (10). 10.1080/17476348.2020.1793673 32635771

[B21] Ferah G. Kutukculer N. (2003). Prospective, randomized comparison of OM-85 BV and a prophylactic antibiotic in children with recurrent infections and immunoglobulin A And/Or G subclass deficiency. Curr. Ther. Res. Clin. Exp. 64 (8). 10.1016/j.curtheres.2003.09.008 PMC4053047 24944407

[B22] Francesco F. Schaub B. (2023). Childhood asthma phenotypes and endotypes: a glance into the mosaic. Mol. Cell Pediatr. 10 (1). 10.1186/s40348-023-00159-1 PMC10469115 37646843

[B23] Gans Melissa D. Gavrilova T. (2019). Understanding the immunology of asthma: pathophysiology, biomarkers, and treatments for asthma endotypes. Paediatr. Respir. Rev. 36 (0), 118–127. 10.1016/j.prrv.2019.08.002 31678040

[B24] Gao Y. Qian X. B. Yu C. Y. (2010). Therapeutic efficacy of bacterial lysates and montelukast treatment in children with intermittent asthma. Chin. J. Clin. Pharmacol. Ther. 15 (5), 539–542.

[B25] Habib N. Pasha M. A. Tang D. D. (2022). Current understanding of asthma pathogenesis and biomarkers. Cells 11 (17), 2764. 10.3390/cells11172764 36078171 PMC9454904

[B26] Han R.-F. Li H.-Y. Wang J.-W. Cong X. J. (2016). Study on clinical effect and immunologic mechanism of infants capillary bronchitis secondary bronchial asthma treated with bacterial lysates broncho-vaxom. Eur. Rev. Med. Pharmacol. Sci. 20 (10), 2151–2155. 27249617

[B27] Higgins J.-P.-T. (2011). Cochrane Handbook for Systematic Reviews of Interventions, 2011. Chichester, United Kingdom: John Wiley and Sons. 14

[B28] Higgins Julian P. T. Thompson S. G. Deeks J. J. Altman D. G. (2003). Measuring inconsistency in meta-analyses. BMJ 327 (7414), 557–560. 10.1136/bmj.327.7414.557 12958120 PMC192859

[B29] Higgins Julian P. T. Altman D. G. Gøtzsche P. C. Juni P. Moher D. Oxman A. D. (2011). The cochrane Collaboration's tool for assessing risk of bias in randomised trials. BMJ 343 (0), d5928. 10.1136/bmj.d5928 22008217 PMC3196245

[B30] Holt P. G. Sly P. D. (2012). Viral infections and atopy in asthma pathogenesis: new rationales for asthma prevention and treatment. Nat. Med. 18 (5), 726–735. 10.1038/nm.2768 22561836

[B31] Hu L. Wen H. (2022). Efficacy of bacterial lysate capsules in adjuvant treatment of children with acute bronchial asthma based on changes in pulmonary function and serum cytokine levels. Rev. Psiquiatr. Clinica 49 (2), 44–48.

[B32] Hu P. L. Luo Y. W. Qian X. B. (2011). Clinical efficacy of fluticasone plus combined with bacterial lysates in treatment of children with asthma. Mod. Chin. Dr. 49 (16).

[B33] Jackson D. J. Gern J. E. (2022). Rhinovirus infections and their roles in asthma: etiology and exacerbations. J. Allergy Clin. Immunol. Pract. 10 (3), 673–681. 10.1016/j.jaip.2022.01.006 35074599 PMC10314805

[B34] Jartti T. Gern J.-E. (2017). Role of viral infections in the development and exacerbation of asthma in children. J. Allergy Clin. Immunol. 140 (4), 895–906. 10.1016/j.jaci.2017.08.003 28987219 PMC7172811

[B35] Jin Q. Li S. F. (2021). Effect of bacterial lysate capsules in adjuvant treatment of children with acute bronchial asthma. Henan Med. Res. 30 (28).

[B36] Joanna Z. Cieślik M. Dumycz K. (2026). Efficacy and safety of bacterial immunostimulants in immunodeficient individuals: a systematic review and meta-analysis. Pediatr. Allergy Immunol. 37 (1). 10.1111/pai.70276 41482727

[B37] José O. M. Marrodan B. R. Prieto Santos N. (2025). Recommendations for the safe use of high-risk medications in pediatrics. Pediatr (Engl Ed) 103 (1). 10.1016/j.anpede.2025.503815 40685171

[B38] Kay W. Stonham C. Rutherford C. (2023). Fractional exhaled nitric oxide (FeNO): the future of asthma care? Br. J. Gen. Pract. 73 (737). 10.3399/bjgp23X735813 PMC10688935 38035806

[B39] Kristina S. Ralph Edwards I. (2014). Pharmacovigilance for children's sake. Drug Saf. 37 (2). 10.1007/s40264-013-0133-8 24446277

[B40] Kuruvilla Merin E. Eun-Hyung Lee F. Lee G. B. (2018). Understanding asthma phenotypes, endotypes, and mechanisms of disease. Clin. Rev. Allergy Immunol. 56 (2), 219–233. 10.1007/s12016-018-8712-1 30206782 PMC6411459

[B41] Lan Li Li J. Hu C. Di Nardo M. Srinivasan V. Adamko D. J. (2022). Effectiveness of polyvalent bacterial lysate for pediatric asthma control: a retrospective propensity score-matched cohort study. Transl. Pediatr. 11 (10), 1697–1703. 10.21037/tp-22-489 36345454 PMC9636455

[B42] Lin M. T. Wang Q. H. Xu B. H. (2023). Clinical effect of bacterial lysate products capsules combined with conventional drugs in the treatment of acute attack of bronchial asthma in children. J. Med. THEORY Pract. 36 (5).

[B43] Lin G. A. O. Zhang H. Chen H.-dan (2024). The therapeutic effect of bacterial dissolution product capsules on children with bronchial asthma and their impact on levels of serum CRP, SAA, PCT, and FeNO. Prog. Mod. Biomed. 24 (13), 2555–2559.

[B44] Liu Y. Q. (2020). The influence of bacterial lysate capsules on serum CD4+/CD8+ levels and airway remodeling in children with bronchial asthma in the remission stage. J. Guangxi Med. Univ. 37 (3), 492–497.

[B45] Lou J. Kong H. Xiang Z. Zhu X. Cui S. Li J. (2025a). The J-shaped association between the ratio of neutrophil counts to prognostic nutritional index and mortality in ICU patients with sepsis: a retrospective study based on the MIMIC database. Front. Cell Infect. Microbiol. 15, 1603104. 10.3389/fcimb.2025.1603104 40766844 PMC12322749

[B46] Lou J. Xiang Z. Zhu X. Song J. Cui S. Li J. (2025b). Association between serum glucose potassium ratio and short- and long-term all-cause mortality in patients with sepsis admitted to the intensive care unit: a retrospective analysis based on the MIMIC-IV database. Front. Endocrinol. (Lausanne) 16, 1555082. 10.3389/fendo.2025.1555082 40810063 PMC12343221

[B47] Lou J. Xiang Z. Zhu X. Song J. Huang N. Li J. (2025c). Oxandrolone for burn patients: a systematic review and updated meta-analysis of randomized controlled trials from 2005 to 2025. World J. Emerg. Surg. 20 (1), 75. 10.1186/s13017-025-00648-w 41023744 PMC12481747

[B48] Lu Y.-M. Li Y.-Q. Xu L.-Y. Xia M. Cao L. (2015). Bacterial lysate increases the percentage of natural killer T cells in peripheral blood and alleviates asthma in children. PHARMACOLOGY 95 (3-4), 139–144. 10.1159/000377683 25833066

[B49] Lu W. D. Zhu M. Yang Y. (2023). The application effect of bacterial lysate capsules combined with nebulization therapy in children with asthma. Chronic Pathematology J. (3), 399–402.

[B50] Luo X. H. Lin D. H. Dai J. Z. (2023). Study on the effect of low-dose fluticasone propionate aerosol inhalation combined with bacterial endotoxin products in the prevention and treatment of recurrent asthmatic diseases in children. Smart Healthc. 9 (10), 126–130.

[B51] Marjolein E. Janssens H. M. De-Jongste J. C. (2014). Medication adherence and the risk of severe asthma exacerbations: a systematic review. Eur. Respir. J. 45 (2). 10.1183/09031936.00075614 25323234

[B52] Muireann N.-C. Lasserson T. J. Greenstone I. (2009). Addition of long-acting beta-agonists to inhaled corticosteroids for chronic asthma in children. Cochrane Database Syst. Rev. (3), CD007697. 10.1002/14651858.CD007949 PMC4167878 19588447

[B53] Page M. J. McKenzie J. E. Bossuyt P. M. (2021). The PRISMA 2020 statement: an updated guideline for reporting systematic reviews. BMJ 372 (0). 10.1136/bmj.n71 PMC8005924 33782057

[B54] Petrisko M. A. Jonathan D. S. Skoner D. P. (2008). Safety and efficacy of inhaled corticosteroids (ICS) in children with asthma. J. Asthma 45 (Suppl 2), 36–42. 10.1080/02770900802631361 19093279

[B55] Picheswara Rao P. Krishna Bikki V. (2025). Asthma endotypes in flux: integrating type 1 and type 2 inflammation for biological therapy advancement. J. Asthma 62 (12). 10.1080/02770903.2025.2555300 40884772

[B56] Qi S. S. Zhang G. W. Meng F. W. (2022). Observation on the effect of capsules of bacterial dissolution products combined with zalusertide and budesonide aerosol in treating children with bronchial asthma in remission period. J. Med. Theory Pract. 35 (23).

[B57] Razi C.-H. Harmanc K. Abac A. Özdemir O. Hızlı Ş. Renda R. (2010). The immunostimulant OM-85 BV prevents wheezing attacks in preschool children. J. Allergy Clin. Immunol. 126 (4), 763–769. 10.1016/j.jaci.2010.07.038 20920766

[B58] Richard H. Farne H. Ritchie A. (2015). The role of viral infections in exacerbations of chronic obstructive pulmonary disease and asthma. Ther. Adv. Respir. Dis. 10 (2). 10.1177/1753465815618113 PMC5933560 26611907

[B59] Richard K. Baty F. Smith H.-J. (2024). Assessment of functional diversities in patients with asthma, COPD, Asthma-COPD overlap, and cystic fibrosis *(cf)* . PLoS One 19 (2). 10.1371/journal.pone.0292270 PMC10878531 38377145

[B60] Sanjiv-Singh Rawat. Global Initiative for Asthma (GINA) (2025). A revolutionary document for management of asthma in children. J. Pediatr. Pulmonol. 4 (2), 29–30.

[B61] Shen P. Xu S. Y. Jiang Y. (2023). The effect of bacterial lysate capsules combined with budesonide aerosol in the treatment of childhood bronchial asthma and its influence on pulmonary function. DA YISHENG 8 (19).

[B62] Sinha Ian P. Gallagher R. Williamson P. R. Smyth R. L. (2012). Development of a core outcome set for clinical trials in childhood asthma: a survey of Clinicians, parents, and young people. Trials 13 (0), 103. 10.1186/1745-6215-13-103 22747787 PMC3433381

[B63] Siri R. Keller T. Icke K. (2020). Orally applied bacterial lysate in infants at risk for atopy does not prevent atopic dermatitis, allergic rhinitis, asthma or allergic sensitization at school age: follow-Up of a randomized trial. Allergy 75 (8). 10.1111/all.14247 32087032

[B64] Solèr M. Mütterlein R. Cozma G. (2006). Double-blind study of OM-85 in patients with chronic bronchitis or mild chronic obstructive pulmonary disease. Respiration 74 (1), 26–32. 10.1159/000093933 16772707

[B65] Sterne J. A. C. Sutton A. J. Ioannidis J. P. A. Terrin N. Jones D. R. Lau J. (2011). Recommendations for examining and interpreting funnel plot asymmetry in meta-analyses of randomised controlled trials. BMJ 343 (0), d4002. 10.1136/bmj.d4002 21784880

[B66] Szefler S. J. (2001). Challenges in assessing outcomes for pediatric asthma. J. Allergy Clin. Immunol. 107 (0), S456–S464. 10.1067/mai.2001.114947 11344375

[B67] Tang Y. Q. Zhao D. H. Sun W. J. (2017). Clinical observation of bacterial lysates capsules in the treatment of acute attack of asthma in children. China Pharm. 28 (32).

[B68] Vadim P. Pivniouk O. DeVries A. Uhrlaub J. L. Michael A. Pivniouk D. (2021). The OM-85 bacterial lysate inhibits SARS-CoV-2 infection of epithelial cells by downregulating SARS-CoV-2 receptor expression. J. Allergy Clin. Immunol. 149 (3), 923–933.e6. 10.1016/j.jaci.2021.11.019 34902435 PMC8660661

[B69] Wang X. (2022). Effects of bacterial lysate in adjuvant therapy of children with bronchial asthma. Med. J. Chin. People's Health 34 (7).

[B70] Wu H. T. (2019). Observation on the therapeutic effect of capsules derived from bacterial dissolution combined with inhaled budesonide suspension in children with acute episode of bronchial asthma. Mod. Diagnosis Treat. 30 (9).

[B71] Xiang W. Wang W. Liu J. (2014). Estimating the sample mean and standard deviation from the sample size, median, range And/or interquartile range. BMC Med. Res. Methodol. 14 (0). 25524443 10.1186/1471-2288-14-135 PMC4383202

[B72] Xu Z. L. Xin X. J. (2022). Effect of aerosol inhalation of broncho-vaxom combined with beclomethasoneon children with acute bronchial asthma attack. J. Math. Med. 35 (2).

[B73] Yaling Yu Li Z. Hu Z. (2025). OM85 ameliorates bleomycin-induced pulmonary fibrosis in mice by inhibiting Notch expression and modulating the IFN-γ/IL-4 ratio. Sci. Rep. 15 (1). 10.1038/s41598-025-89874-5 PMC11825818 39948140

[B74] Yang F. (2017). Effect of broncho-Vaxom combined with atomization inhaled budesonide on T lymphocytes in acute attack of child bronchial asthma. Laboratory Med. Clin. 14 (11).

[B75] Yang S. B. (2020). Effect of bacterial lysates combined with salmeterol-fluticasone powder inhalation in bronchial asthma. Chin. J. Microecology 32 (6).

[B76] Yang F. Hua L. Liu H. P. (2020a). Therapeutic efficacy of bacterial lysates prevents in children with asthma. J. Clin. Exp. Med. 19 (2).

[B77] Yang L. W. Zhang Y. L. Liu X. (2020b). Clinical effect of immunomodulator on acute exacerbations of bronchial asthma in children and its influence on immune Function,ECP and FENO. Clin. Misdiagnosis and Mistherapy 33 (2).

[B78] Zhang Z. Y. (2021). Study on the effect of broncho-vaxom combined with budesonide on respiratory function and serum IL-4 and IL-10 levels in children with asthma. Med. J. Liaoning 35 (1).

[B79] Zhang H. Ding D. (2019). The clinical efficacy of oxygen-driven nebulized inhalation of glucocorticoids combined with bacterial lysate capsules in the treatment of children with acute exacerbation of bronchial asthma. Med. Equip. 32 (14).

[B80] Zhang S. L. Gu Y. X. Hu J. Y. (2014). The effects of bacterial lysate capsules on pediatric asthma and serum levels of IL4, IFN-γ, and IgE. J. Guiyang Med. Coll. 39 (1), 54–56.

[B81] Zhu Z. J. (2021). Efficacy evaluation of bacterial lysate capsules combined with budesonide suspension atomization in the treatment of children with bronchial asthma. Drug Eval. 18 (16).

[B82] Zhuang-Gui C. Ji J.-Z. Li M. (2007). Immunoregulants improves the prognosis of infants with wheezing. Nan Fang. Yi Ke Da Xue Xue Bao 27 (10). 17959549

