# Supplementary material for: Impact of oral bacterial lysates on asthma control and immune parameters in children: evidence from an updated systematic review and meta-analysis of randomized trials
Source: Front Pharmacol. 2026 Jun 11;17:1801312. doi: 10.3389/fphar.2026.1801312 (PMC13293580; doi:10.3389/fphar.2026.1801312)
Supplement: Supplementary file 2 [file Table1.docx]

# Search Logic: Population (pediatric asthma) + Intervention (oral bacterial lysates) + Study Type (randomized controlled trials) (("Asthma"[MeSH Terms] OR "Asthma"[All Fields] OR "Bronchial Asthma"[All Fields]) AND ("Child"[MeSH Terms] OR "Children"[All Fields] OR "Adolescent"[MeSH Terms] OR "Adolescents"[All Fields] OR "Pediatric"[All Fields] OR "Paediatric"[All Fields] OR "Infant"[MeSH Terms] OR "Infants"[All Fields])) AND (("Bacterial Lysates"[All Fields] OR "Bacterial Lysate"[All Fields] OR "OM-85"[All Fields] OR "Broncho-Vaxom"[All Fields] OR "FanFuShu"[All Fields] OR "Polyvalent Mechanical Bacterial Lysate"[All Fields] OR "PMBL"[All Fields] OR "Ismigen"[All Fields] OR "Oral Immunomodulator"[All Fields])) AND (("Randomized Controlled Trial"[MeSH Terms] OR "RCT"[All Fields] OR "Randomized"[All Fields] OR "Randomly Assigned"[All Fields]))

Key Notes

1. Search Timeframe: From database inception to February 28, 2025 (consistent with the main manuscript).

2. Language Restriction: None. Both English and Chinese studies were included, with no language filter applied during search (screened via eligibility criteria afterward).

3.Search Fields: Combined MeSH Terms (Medical Subject Headings) and All Fields to ensure high recall and avoid missing relevant studies.

4. Term Explanation: "FanFuShu" is the Chinese generic name for OM-85; "PMBL/Ismigen" refers to the other core intervention, both included to cover all target treatments.

5. Study Type Restriction: Limited to randomized controlled trials via MeSH Term "Randomized Controlled Trial" and free-text terms like "RCT", aligning with the manuscript’s inclusion criteria.
